# Protective effects of exogenous melatonin therapy against oxidative stress to male reproductive tissue caused by anti-cancer chemical and radiation therapy: a systematic review and meta-analysis of animal studies

**DOI:** 10.3389/fendo.2023.1184745

**Published:** 2023-08-28

**Authors:** Niloofar Dehdari Ebrahimi, Alireza Sadeghi, Sara Shojaei-Zarghani, Mohammad Amin Shahlaee, Erfan Taherifard, Zahra Rahimian, Zahra Eghlidos, Negar Azarpira, Ali Reza Safarpour

**Affiliations:** ^1^ Transplant Research Center, Shiraz University of Medical Sciences, Shiraz, Iran; ^2^ Gastroenterohepatology Research Center, Shiraz University of Medical Sciences, Shiraz, Iran; ^3^ Colorectal Research Center, Shiraz University of Medical Sciences, Shiraz, Iran; ^4^ MPH Department, Medical School, Shiraz University of Medical Sciences, Shiraz, Iran; ^5^ Department of Medicine, Shiraz University of Medical Sciences, Shiraz, Iran

**Keywords:** rodents, melatonin, male reproduction, testicular tissue, cancer, radiotherapy, chemotherapy

## Abstract

**Background:**

Male testicular dysfunction is a considerable complication of anti-cancer therapies, including chemotherapy and radiotherapy, partly due to the increased oxidative stress caused by these treatments. Melatonin is an effective antioxidant agent that protects testicles against physical and toxic chemical stressors in animal models. This study aims to systematically review the melatonin’s protective effects against anti-cancer stressors on rodential testicular tissue.

**Materials and Method:**

An extensive search was conducted in Web of Science, Scopus, and PubMed for animal studies investigating exogenous melatonin’s protective effects on rodent testicles exposed to anti-cancer chemicals and radiotherapeutic agents. Using the DerSimonian and Laird random-effect model, standardized mean differences and 95% confidence intervals were estimated from the pooled data. The protocol was prospectively registered in the International Prospective Register of Systematic Reviews (PROSPERO: CRD42022355293).

**Results:**

The meta-analysis included 38 studies from 43 studies that were eligible for the review. Rats and mice were exposed to radiotherapy (ionizing radiations such as gamma- and roentgen radiation and radioactive iodine) or chemotherapy (methotrexate, paclitaxel, busulfan, cisplatin, doxorubicin, vinblastine, bleomycin, cyclophosphamide, etoposide, Taxol, procarbazine, docetaxel, and chlorambucil). According to our meta-analysis, all outcomes were significantly improved by melatonin therapy, including sperm quantity and quality (count, motility, viability, normal morphology, number of spermatogonia, Johnsen’s testicular biopsy score, seminiferous tubular diameter, and seminiferous epithelial height), serum level of reproductive hormones (Follicle-Stimulating Hormone and testosterone), tissue markers of oxidative stress (testicular tissue malondialdehyde, superoxide dismutase, glutathione peroxidase, catalase, glutathione, caspase-3, and total antioxidant capacity), and weight-related characteristics (absolute body, epididymis, testis, and relative testis to body weights). Most SYRCLE domains exhibited a high risk of bias in the included studies. Also, significant heterogeneity and small-study effects were detected.

**Conclusion:**

In male rodents, melatonin therapy was related to improved testicular histopathology, reproductive hormones, testis and body weights, and reduced levels of oxidative markers in testicular tissues of male rodents. Future meticulous studies are recommended to provide a robust scientific backbone for human applications.

**Systematic review registration:**

https://www.crd.york.ac.uk/prospero/display_record.php?ID=CRD42022355293, identifier CRD42022355293.

## Graphical Abstract

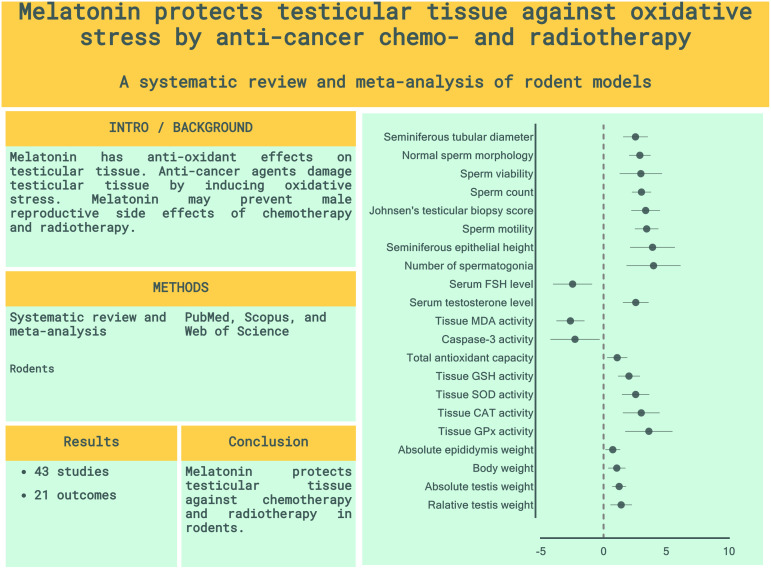

## 1 Introduction

Cancer is the second dominant cause of death globally. In 2020, 19.3 million new patients with cancer were diagnosed, and 10 million deaths associated with cancer were detected worldwide (1). Radio- and chemotherapy are among the most common treatments for malignancies. These strategies are considered double-edged swords, which exert unwanted side effects on healthy tissues, including the male reproductive system. Radiotherapy and chemotherapy could cause male testicular dysfunction, partly by increasing testicular oxidative stress and subsequently inducing lipid peroxidation, DNA damage, mitochondrial dysfunction, and apoptosis (2, 3). These therapeutic methods could also trigger endoplasmic reticulum (ER) stress and inflammation in the testes, leading to cell death and potentially impairing male fertility (2, 4, 5). Differentiating spermatogonia cells are more sensitive than spermatocytes, spermatids, and Leydig cells, which produce testosterone, to the mentioned cytotoxic effects (6, 7). Radio- and chemotherapy are known to cause several reproductive impairments in males, including but not limited to a decrease in sperm count (oligozoospermia), absence of sperm in the ejaculate (azoospermia), morphological abnormalities in spermatozoa (teratozoospermia), low sperm motility (asthenozoospermia), and reduced sperm viability. These effects may persist for an extended period, possibly lifelong (3, 8). Furthermore, undergoing cancer treatments can lead to reduced testosterone levels, as well as compensatory damage to the hypothalamic-pituitary-gonadal axis and Sertoli cells (2, 9). With dramatically increased survival rates, especially in patients of younger ages, reducing the side effects of anti-cancer therapies and preserving fertility can improve their quality of life.

Melatonin is secreted naturally by the pineal gland and is known for its functions in circadian rhythms. Additionally, research is being conducted to evaluate its effects on various diseases, including cancer, cardiovascular disease, and metabolic disorders (10). Also, melatonin membrane receptors (MT1 and MT2) are detectable in several testicular cells, including Sertoli cells, Leydig cells, and germ cells (11), which suggest fundamental roles in the optimal reproductive function in the physiologic conditions (11–13). Decreased serum melatonin levels and downregulation of its receptors are reported following chemotherapy treatments (14–16). The administration of melatonin has been suggested as a potential protective measure against the adverse effects of radiotherapy and chemotherapy on multiple organs, including the brain, heart, kidney, liver, and intestine. This protective effect is thought to be mediated by various mechanisms, such as anti-inflammatory, antioxidant, anti-nitrosative, anti-apoptotic, immune regulatory, and antioxidant defense system-related gene expression regulatory properties (17, 18).

Several studies have investigated melatonin’s protective properties against radiotherapy and chemotherapy-induced injuries on the male reproductive system (19–23). However, no meta-analysis study has reported the net effects and discussed the underlying mechanism. Therefore, we aimed to assess the impact of melatonin on radiotherapy- and chemotherapy-induced male reproductive dysfunction and shed light on the underlying mechanisms.

## 2 Materials and methods

This systematic review and meta-analysis was designed based on the Preferred Reporting Items for Systematic Reviews and Meta-analyses guideline (PRISMA) (24). Prospective protocol registration was done at the International Prospective Register of Systematic Reviews (PROSPERO: CRD42022355293).

### 2.1 Data sources and search

A comprehensive search strategy was developed using “melatonin” and “reproductive indices” and related terms. Three online databases (Web of Science, Scopus, and PubMed) were searched for studies published since January 1^st^, 1970, until September 9^th^, 2022. Moreover, to include additional studies, a manual backward and forward citation search was conducted for all included studies. The search strategy and syntax details are exhibited in **Supplementary Material 1**.

### 2.2 Study selection

The duplicate records were removed and uploaded to the Rayyan web-based tool for systematic review management (25). Three reviewers (NDE, ET, and MAS) screened the records independently by titles and abstracts. Then, full texts were retrieved for each study for screening by eligibility criteria. Disagreements were resolved through discussion.

Studies were considered eligible to include if they met the following criteria: (a) controlled animal studies, (b) included male rodents who were exposed to anti-cancer chemo- or radiotherapy agents, (c) in at least one intervention arm, melatonin was administered, (d) one or more positive control arms (with or without placebo), (e) The major characteristics of testicular tissue have been reported (sperm analyses, biochemical, and histopathologic). Studies were excluded if they had (a) ex-vivo and *in-vitro* designs, (b) non-rodent subjects, (c) stressors other than conventional anti-cancer chemo- and radiotherapy, and (d) a combination of melatonin and other drugs was administered. Furthermore, human trials, letters, and reviews were excluded from this review. We did not apply any restrictions based on the language or date of publication.

### 2.3 Data extraction and assessment of the risk of bias

Data extraction was performed into an Excel spreadsheet by four reviewers (NDE, NE, ZR, and MAS). The differences were resolved by discussion. Based on the results of each study, the following outcomes were extracted (if available): (a) study characteristics (first author, country, and publication year), (b) subject characteristics (sample size, age, and species), (c) chemical or radiation agent and their dosages, route of administration, and duration of exposure, (d) melatonin’s dosage, duration of therapy, administration route, and timing of administration relative to stressor, (e) sperm-related characteristics (count, motility, viability, normal morphology, number of spermatogonia, seminiferous epithelial height, Johnsen’s testicular biopsy score (JTBS), and seminiferous tubular diameter), (f) serum reproductive hormone levels (Follicle-Stimulating Hormone (FSH) and testosterone), (g) tissue oxidative stress markers (glutathione (GSH), Catalase (CAT), testicular tissue Superoxide dismutase (SOD), Malondialdehyde (MDA), glutathione peroxidase (GPx), Caspase-3, and Total Antioxidant Capacity (TAC)), and (h) weight-related characteristics (absolute body, testis, epididymis, and relative testis to body weights).

Based on the Systematic Review Centre for Laboratory Animal Experiments (SYRCLE) tool for animal intervention studies, the risk of bias was assessed independently by two reviewers (AS and NDE) (26).

### 2.4 Data synthesis and statistical analysis

Data were analyzed using Stata MP Version 16 (StataCorp, College Station, TX, USA), and a p-value <0.05 was considered statistically significant. Based on the DerSimonian-Laird method, a random effect model was utilized to pool the effect sizes using Standardized Mean Difference (SMD) for meta-analyses. Also, a 95% confidence interval (CI) was reported for each effect size. The residual heterogeneity between studies was evaluated using the Cochran’s Q statistic, I-squared, and p-value. I-squared was interpreted as “perhaps not important”, “moderate heterogeneity”, “substantial heterogeneity”, and “considerable heterogeneity” when values were 0-40%, 30-60%, 50-90%, and 75-100%, respectively (27). Multiple intervention arms were combined using Cochrane’s formula to avoid overcalculations in the studies with shared control groups (27). To identify potential sources of heterogeneity, subgroup analyses were applied only in cases of three or more available studies per subgroup. Also, to obtain missing data, reviewers tried to reach the authors via email and waited for at least one month for responses. Studies were removed from the analyses if their missing data were crucial. Also, when minimum, median, quartiles, and maximum were the only available statistics, mean and standard deviation were estimated using previously published statistical methods (28, 29). Furthermore, funnel plots were developed for outcomes with more than ten studies (27). Visual inspection for asymmetry and Egger’s regression test for small-study effects were done to detect publication bias (30).

## 3 Results

### 3.1 Search results

**Figure 1:**
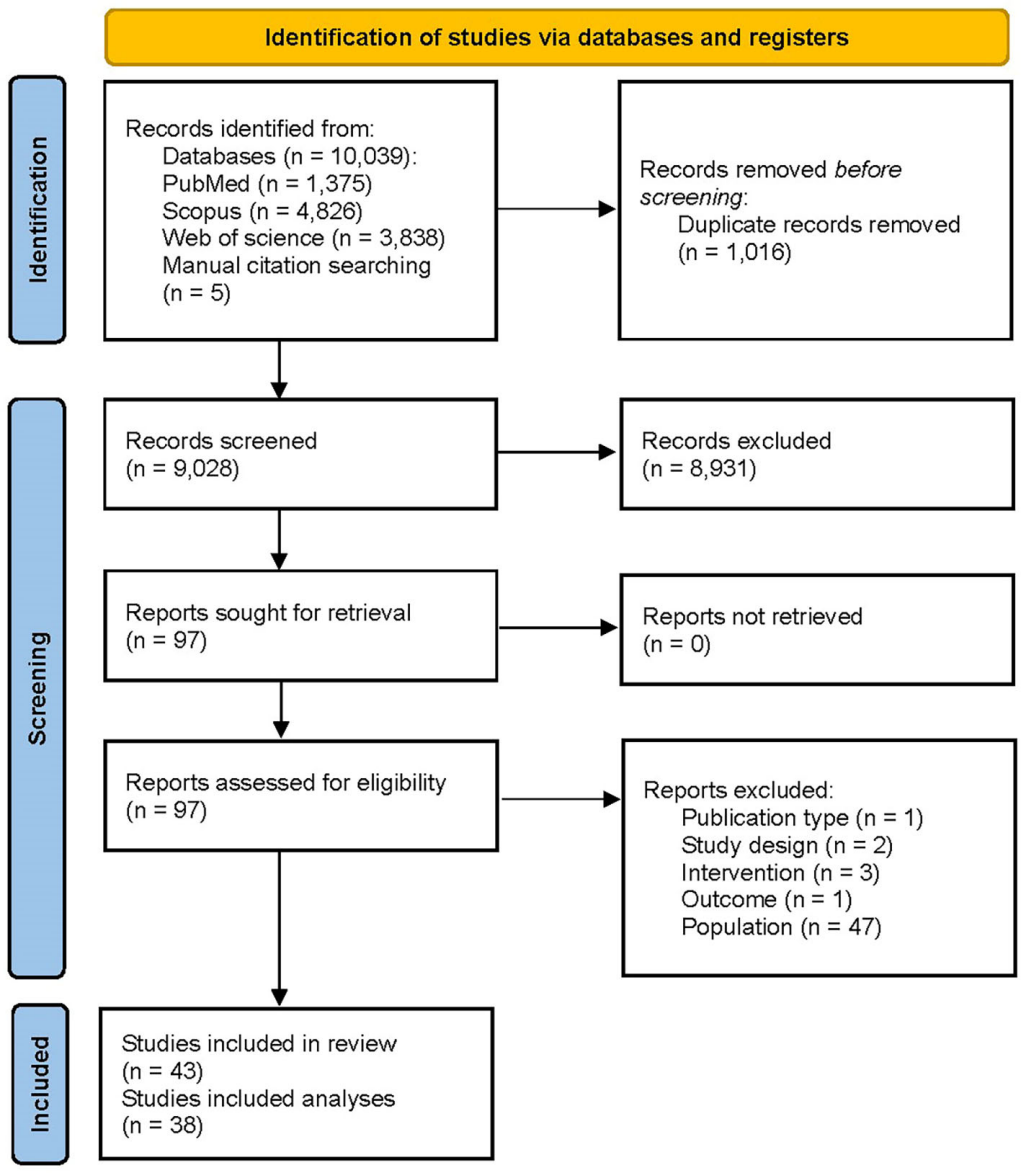
PRISMA flow diagram illustrating the process of selection of the studies.

A total of 10,039 and 5 records were obtained from the systematic database and manual citation searching, respectively. The title and abstract of 9,028 unique documents were screened after omitting 1,016 duplicate records. 97 articles were checked for eligibility, and a final 43 articles were included in the systematic review. Among the included studies, 5 (21, 31–34) were only included in the narrative evidence synthesis, and 38 were used in the meta-analyses. The PRISMA flow diagram is presented in **Figure 1**.

### 3.2 Study characteristics

Included studies were published between 2003 and 2023 in English (n=36) (14, 19–22, 31–66) and Persian (n=2) (67, 68). The studies were published from Iran (n=11) (20, 40, 45, 53–55, 59, 61, 65, 67, 68), Turkey (n=9) (21, 31, 36–39, 43, 47, 64), Egypt (n=9) (33–35, 41, 42, 44, 46, 48, 60), China (n=7) (14, 19, 32, 49, 62, 63, 66), India (n=4) (22, 50, 52, 57), Thailand (n=1) (58), Nigeria (n=1) (56), and South Korea (n=1) (51). Studies employed rats (n=25) (20–22, 31, 34–37, 39, 41–44, 46–48, 51, 56–58, 61, 62, 64, 65, 68) and mice (n=18) (14, 19, 32, 33, 38, 40, 45, 49, 50, 52–55, 59, 60, 63, 66, 67) as subjects. To induce stress, the included studies employed ionizing radiations (n=9) (21, 39, 46, 48–50, 59, 60, 64) and chemical agents (n=34) (14, 19, 20, 22, 31–38, 40–45, 47, 51–58, 61–63, 65–68). For chemical therapy, methotrexate (58, 62), paclitaxel (63), busulfan (19, 32–34, 45, 53–55, 65, 67, 68), cisplatin (14, 20, 22, 37, 40–44, 47), doxorubicin (51, 57, 66), vinblastine (22), bleomycin (20, 22), cyclophosphamide (31, 47, 52, 61), etoposide (20), Taxol (35), procarbazine (36), docetaxel (38), and chlorambucil (56) were employed. Melatonin was administered intraperitoneal (IP, n=32) (14, 19–22, 31–41, 43, 45–47, 49, 50, 53–55, 58–61, 63–68) and oral (n=8) (42, 44, 48, 51, 52, 56, 57, 62). Detailed study characteristics, including stressor and melatonin dosages, duration of exposure to each one, and number and age of rodents, are provided in **Table 1** and **Supplementary Material 2**.

**Table 1:** Basic characteristics of the included studies.

First author [year]	Country	Rodent	Age of subjects	Number of subjects (intervention/control)	Model of intervention	Type of OS	SYRCLE score
**Wang [2018] **(62)	China	Rats	N/M	8/8	Preventive	Chemical agent (Methotrexate)	2
**Wang [2022] **(63)	China	Mice	8 weeks	10/10	Therapeutic	Chemical agent (Paclitaxel)	3
**Yalcınkaya [2009] (poster) **(64)	Turkey	Rats	10-12 weeks	10/10	Therapeutic	Radiation (Gamma radiation)	N/A
**Zangoie [2019]** (65)	Iran	Rats	N/M	6/6	Therapeutic	Chemical agent (Busulfan)	2
**Zhang [2022]** (14)	China	Mice	8 weeks	8/8	Therapeutic	Chemical agent (Cisplatin)	4
**Zi [2022]** (66)	China	Mice	6-8 weeks	5/5	Preventive	Chemical agent (Doxorubicin)	3
**Hussein [2006] **(46)	Egypt	Rats	3 months	20/20	Preventive	Radiation (Roentgen radiation)	2
**Khan [2015] **(49)	China	Mice	8-9 weeks	3/3	Preventive	Radiation (Gamma radiation)	2
**Kushwaha [2021] **(50)	India	Mice	8-10 weeks	6/6	Preventive	Radiation (Gamma radiation)	2
**Lee [2012]** (51)	South Korea	Rats	8 weeks	6/6	Preventive	Chemical agent (Doxorubicin)	3
**Madhu [2015] **(22)	India	Rats	N/M	8/8	Preventive	Chemical agent (Cisplatin + Vinblastine + Bleomycin)	4
**Manda [2003] **(52)	India	Mice	6-8weeks	10/10	Preventive	Chemical agent (Cyclophosphamide)	3
**Mirhoseini [2014] **(53)	Iran	Mice	6-7 weeks	7/7	Therapeutic	Chemical agent (Busulfan)	3
**Taheri Moghadam [2021]** (54)	Iran	Mice	4-6 weeks	6/6	Preventive	Chemical agent (Busulfan)	3
**Moradi [2021]** (20)	Iran	Rats	13-15 weeks	5/5	Preventive	Chemical agent (Bleomycin + Etoposide + Cisplatin)	3
**Cebi Sen [2018]** (39)	Turkey	Rats	N/M	12/12	Preventive	Radiation (Radioactive iodine)	3
**Patil [2009]** (57)	India	Rats	N/M	6/6	Preventive	Chemical agent (Doxorubicin)	2
**Aboelwafa [2022]** (35)	Egypt	Rats	16-18 weeks	5/5	Therapeutic	Chemical agent (Taxol)	4
**Alp [2014]** (36)	Turkey	Rats	N/M	6/8	Preventive	Chemical agent (Procarbazine)	2
**Atessahin [2006]** (37)	Turkey	Rats	8 weeks	6/6	Preventive	Chemical agent (Cisplatin)	3
**Baş [2019]** (38)	Turkey	Mice	6-8 weeks	8/8	Therapeutic	Chemical agent (Docetaxel)	3
**Chabra [2014]** (40)	Iran	Mice	N/M	5/5	Preventive	Chemical agent (Cisplatin)	2
**Cui [2017]** (19)	China	Mice	8 weeks	3/3	Therapeutic	Chemical agent (Busulfan)	3
**Edrees [2012]** (41)	Egypt	Rats	N/M	5/5	Preventive	Chemical agent (Cisplatin)	2
**Kamal El-Dein [2020]** (48)	Egypt	Rats	N/M	6/6	Therapeutic	Radiation (Gamma radiation)	3
**El-Shafaei [2018]** (42)	Egypt	Rats	N/M	10/10	Therapeutic	Chemical agent (Cisplatin)	3
**Yilmaz [2019]** (43)	Turkey	Rats	3-5 months	8/8	Preventive	Chemical agent (Cisplatin)	3
**Filobbos [2020]** (44)	Egypt	Rats	12 weeks	10/10	Preventive	Chemical agent (Cisplatin)	3
**Mohamad Ghasemi [2010] (i) **(45)	Iran	Mice	N/M	6/6	Therapeutic	Chemical agent (Busulfan)	3
**Ilbey [2008]** (47)	Turkey	Rats	6 weeks	6/6	Preventive	Chemical agent (Cisplatin and Cyclophosphamide)	3
**Mohamad Ghasemi [2010] (ii)** (55)	Iran	Mice	6-8weeks	8/8	Therapeutic	Chemical agent (Busulfan)	2
**Ferdosi Khosroshahi [2013] (Farsi)** (68)	Iran	Rats	N/M	10/10	Therapeutic	Chemical agent (Busulfan)	2
**Mohammd Ghasemi [2009] (Farsi)** (67)	Iran	Mice	6-8 weeks	8/8	Therapeutic	Chemical agent (Busulfan)	2
**Olayaki [2019] **(56)	Nigeria	Rats	N/M	10/10	Therapeutic	Chemical agent (Chlorambucil)	2
**Tawfik [2006]** (60)	Egypt	Mice	7-9 weeks	6/6	Preventive	Radiation (Gamma radiation)	3
**Sukhorum [2020] **(58)	Thailand	Rats	N/M	8/8	Preventive	Chemical agent (Methotrexate)	2
**Tajabadi [2020] **(59)	Iran	Mice	6-8 weeks	5/5	Therapeutic	Radiation (Gamma radiation)	2
**Torabi [2017]** (61)	Iran	Rats	6-8 weeks	7/7	Preventive	Chemical agent (Cyclophosphamide)	3
**Take [2009]** (21)	Turkey	Rats	6-7 weeks	32/32	Preventive	Ionizing irradiation	2
**Zhang [2019]** (32)	China	Mice	3 weeks	20/20	Preventive	Chemical agent (Busulfan)	3
**Abou-El-Naga [2021]** (33)	Egypt	Mice	N/M	5/5	Therapeutic	Chemical agent (Busulfan)	3
**Abd-El-Aziz [2012] **(34)	Egypt	Rats	N/M	10/7	Therapeutic	Chemical agent (Busulfan)	3
**Simsec [2008] **(31)	Turkey	Rats	5-6 weeks	5/5	Preventive	Chemical agent (Cyclophosphamide)	2

OS, oxidative stress; N/M, not mentioned; N/A, not applicable; SYRCLE, Systematic Review Centre for Laboratory Animal Experimentation.

### 3.3 Outcomes

**Figure 2:**
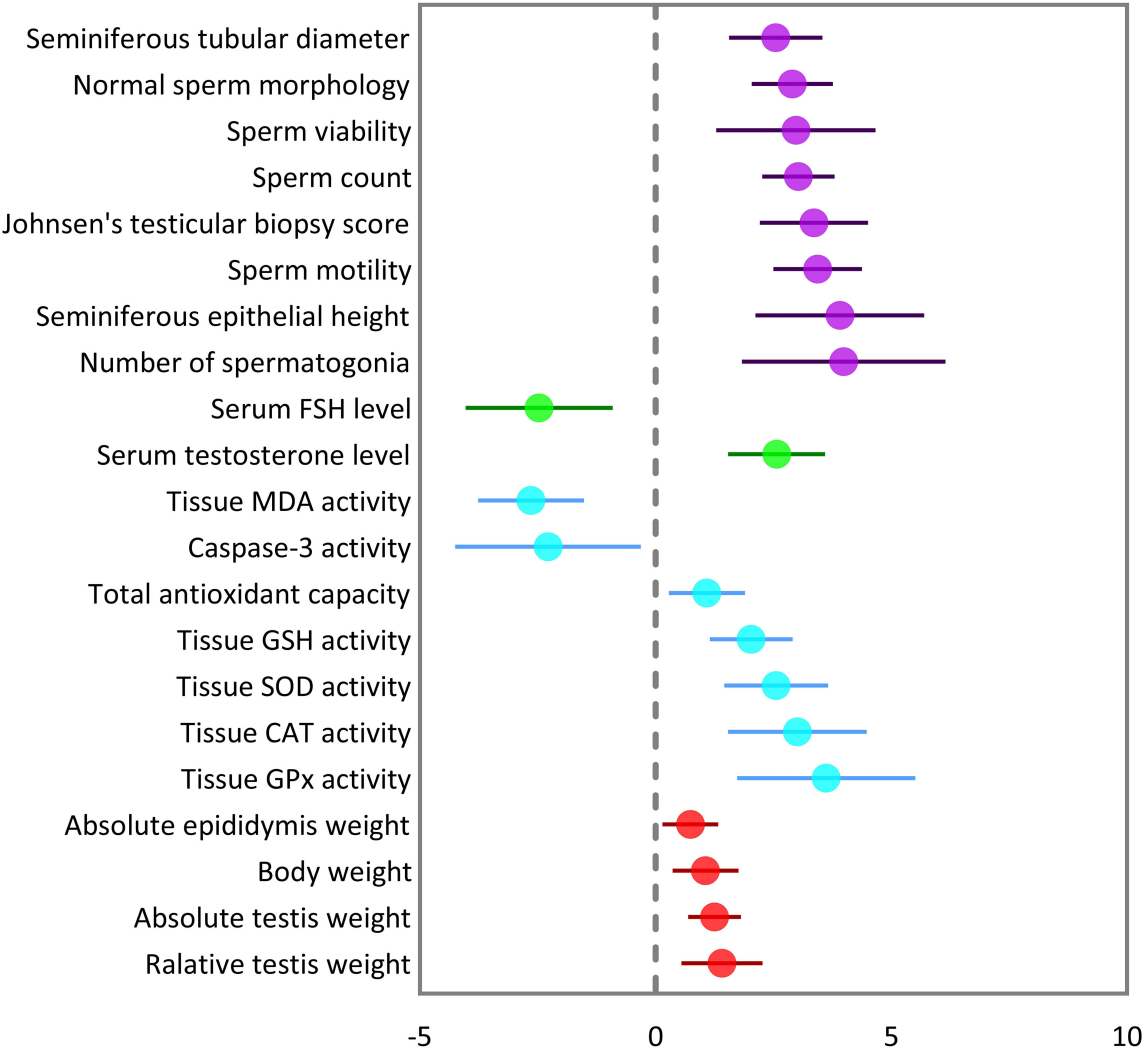
Summary of overall pooled effect sizes for each outcome. Sperm-related parameters are indicated in purple, reproductive hormones in green, oxidative markers of testicular tissue in cyan, and body and testicular weights in red.

**Figure 3:**
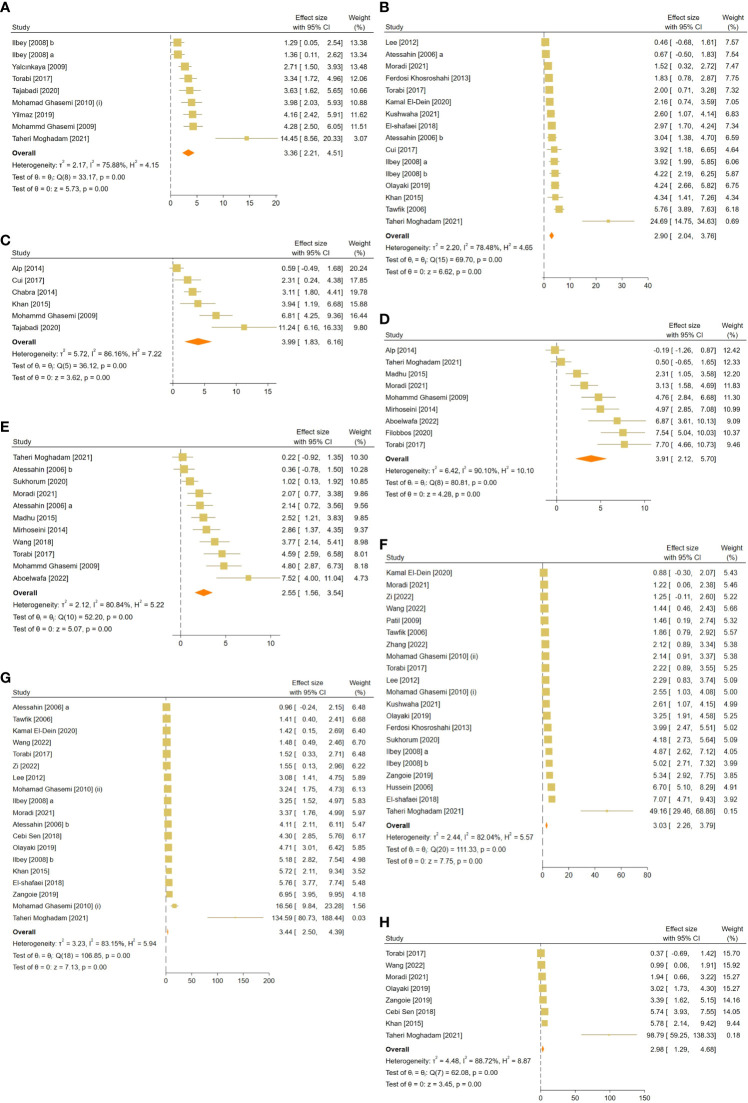
Forest plots for the overall pooled effects sizes of sperm-related parameters including **(A)** JTBS, **(B)** normal sperm morphology, **(C)** number of spermatogonia, **(D)** seminiferous epithelial height, **(E)** seminiferous tubular diameter, **(F)** sperm count, **(G)** sperm motility, and **(H)** sperm viability. JTBS, Johnsen’s testicular biopsy score.

**Figure 4:**
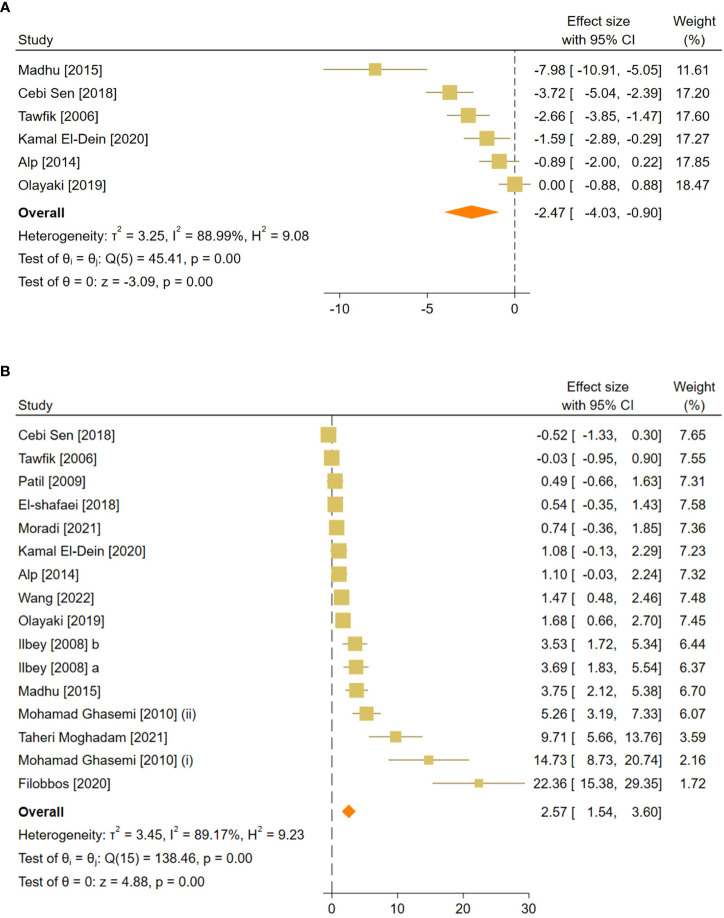
Forest plots for the overall pooled effects sizes of reproductive hormones, including **(A)** serum FSH and **(B)** testosterone level. FSH, Follicle-Stimulating Hormone.

**Figure 5:**
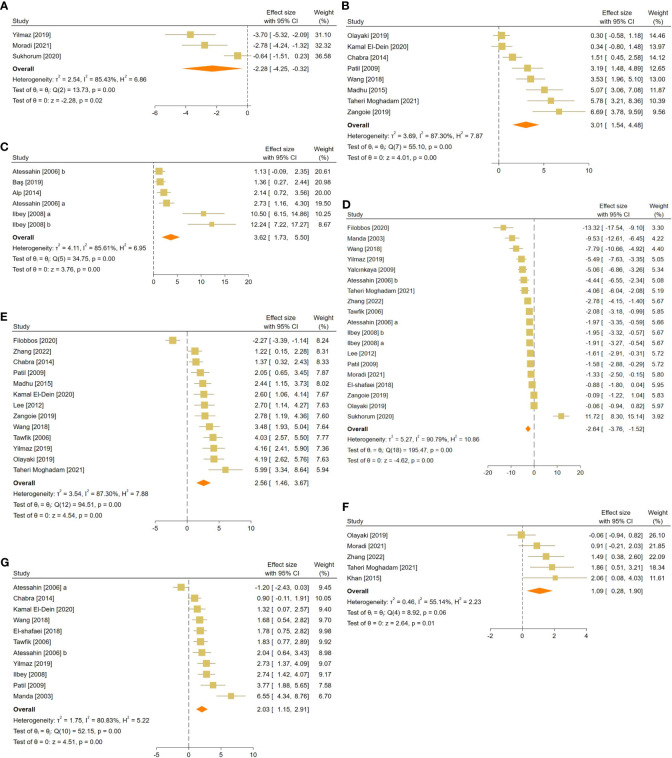
Forest plots for the overall pooled effects sizes of testicular tissue’s oxidative markers, including **(A)** caspase-3, **(B)** tissue catalase, **(C)** glutathione peroxidase, **(D)** malondialdehyde, **(E)** superoxide dismutase activity, **(F)** total antioxidant capacity, and **(G)** glutathione activity.

**Figure 6:**
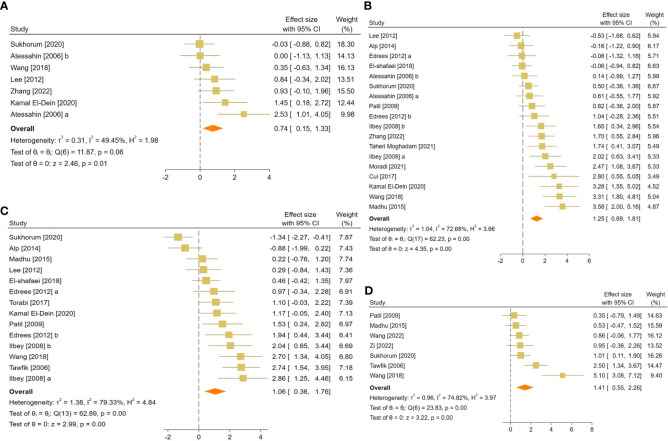
Forest plots for the overall pooled effects sizes of body and testicular weights, including **(A)** absolute epididymis weight, **(B)** absolute testis weight, **(C)** body weight, and **(D)** testis to body relative weight.

The pooled SMDs were statistically significant for all of the 21 outcomes. The outcomes were classified into four categories: (a) sperm-related parameters, (b) reproductive hormones, (c) markers of oxidative stress and apoptosis in testicular tissue, and (d) body and testicular weights. The pooled outcomes included absolute epididymis, testis, and body weights, testis to body relative weight, caspase-3 activity, tissue CAT, GPX, MDA, SOD, and GSH activity, TAC, serum FSH and testosterone levels, JTBS, normal sperm morphology, number of spermatogonia, seminiferous epithelial height, seminiferous tubular diameter, sperm count, motility, and viability. The overall pooled effect sizes for each outcome are summarized in the **Figure 2**. Detailed forest plots of the overall pooled effects sizes for each outcome are presented in **Figures 3**–**6**.

#### 3.3.1 Sperm-related parameters

The pooled SMDs for each sperm-related parameter were: JTBS (SMD = 3.36, 95% CI: 2.21 to 4.51, p-value <0.01), normal sperm morphology (SMD = 2.9, 95% CI: 2.04 to 3.76, p-value <0.01), number of spermatogonia (SMD = 3.99, 95% CI: 1.83 to 6.16, p-value <0.01), seminiferous epithelial height (SMD = 3.91, 95% CI: 2.12 to 5.7, p-value <0.01), seminiferous tubular diameter (SMD = 2.55, 95% CI: 1.56 to 3.54, p-value <0.01), sperm count (SMD = 3.03, 95% CI: 2.26 to 3.79, p-value <0.01), motility (SMD = 3.44, 95% CI: 2.5 to 4.39, p-value <0.01), and viability (SMD = 2.98, 95% CI: 1.29 to 4.68, p-value <0.01). Between-study heterogeneity was substantial to considerable for sperm-related parameters with JTBS (I^2 = ^75.88% and p-value for Q test <0.01), normal sperm morphology (I^2 = ^78.48% and p-value for Q test <0.01), number of spermatogonia (I^2 = ^86.16% and p-value for Q test <0.01), seminiferous epithelial height (I^2 = ^90.1% and p-value for Q test <0.01), seminiferous tubular diameter (I^2 = ^80.84% and p-value for Q test <0.01), sperm count (I^2 = ^82.04% and p-value for Q test <0.01), motility (I^2 = ^83.15% and p-value for Q test <0.01), and viability (I^2 = ^88.72% and p-value for Q test <0.01).

#### 3.3.2 Reproductive hormones

The combined SMDs for serum FSH and testosterone levels were (SMD = -2.47, 95% CI: -4.03 to -0.9, p-value <0.01) and (SMD = 2.57, 95% CI: 1.54 to 3.6, p-value <0.01), respectively. Between-study heterogeneity was considerable for serum reproductive hormone levels with FSH (I^2 = ^88.9% and p-value for Q test <0.01) and testosterone (I^2 = ^89.17% and p-value for Q test <0.01).

#### 3.3.3 Testicular tissue’s oxidative markers

For each oxidative marker, the pooled SMDs were as follows: caspase-3 (SMD = -2.28, 95% CI: -4.25 to -0.32, p-value = 0.02), tissue CAT (SMD = -2.28, 95% CI: -4.25 to -0.32, p-value = 0.02), GPX (SMD = 3.62, 95% CI: 1.73 to 5.5, p-value <0.01), MDA (SMD = -2.64, 95% CI: -3.76 to -1.52, p-value <0.01), SOD (SMD = 2.56, 95% CI: 1.46 to 3.67, p-value <0.01), and GSH (SMD = 2.03, 95% CI: 1.15 to 2.91, p-value <0.01) activity, and TAC (SMD = 1.09, 95% CI: 0.28 to 1.9, p-value = 0.01). Between-study heterogeneity was considerable for oxidative markers of testicular tissue with caspase-3 (I^2 = ^85.43% and p-value for Q test <0.01), tissue CAT (I^2 = ^87.3% and p-value for Q test <0.01), GPX (I^2 = ^85.61% and p-value for Q test <0.01), MDA (I^2 = ^90.79% and p-value for Q test <0.01), SOD (I^2 = ^87.3% and p-value for Q test <0.01), and GSH (I^2 = ^80.83% and p-value for Q test <0.01) activity, and TAC (I^2 = ^55.14% and p-value for Q test 0.06).

#### 3.3.4 Body and testicular weights

The pooled SMDs were absolute epididymis (SMD = 0.74, 95% CI: 0.15 to 1.33, p-value = 0.01), testis (SMD = 1.25, 95% CI: 0.69 to 1.81, p-value <0.01), and body weights (SMD = 1.06, 95% CI: 0.36 to 1.76, p-value <0.01), and testis to body relative weight (SMD = 1.41, 95% CI: 0.55 to 2.26, p-value <0.01). Body and testicular weights showed moderate to substantial heterogeneity between studies with absolute epididymis (I^2 = ^49.45% and p-value for Q test 0.06), testis (I^2 = ^78.68% and p-value for Q test <0.01), and body weights (I^2 = ^79.33% and p-value for Q test <0.01), and testis to body relative weight (I^2 = ^74.82% and p-value for Q test <0.01).

### 3.4 Subgroup analyses

The subgroup analyses were conducted on rodent species (mice versus rats), timing of intervention (preventive versus therapeutic, respectively, indicating melatonin therapy was started before and after the induction of stress), route of administration of melatonin, and type of stressor (chemical versus radiation). Subgroup analyses failed to indicate the source of heterogeneity. However, significant between-group differences were observed between the relative timing of intervention for serum FSH level and rodent species for JTBS and normal sperm morphology and count. The forest plots for subgroup analyses are provided in the **Supplementary Material 3**.

### 3.5 Sensitivity analyses and risk of bias assessment

The results’ robustness was assessed using the leave-one-out method. After removing each study from the analyses, the pooled effect sizes did not significantly change. The forest plots for sensitivity analyses for each outcome are provided in the **Supplementary Material 4**.

**Figure 7:**
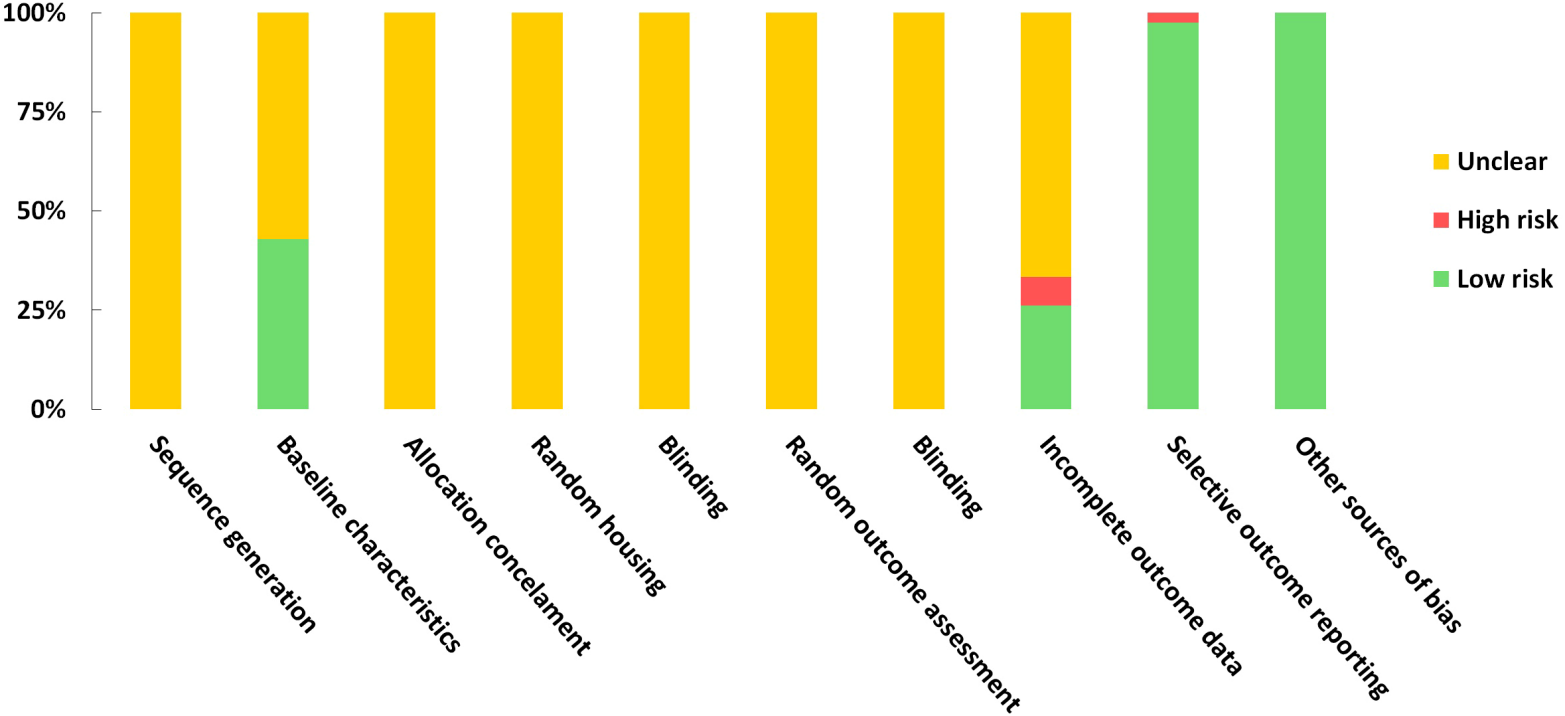
Risk of bias graph on judgements about each risk of bias item presented as proportions across all the included studies.

A risk of bias assessment was conducted using the SYRCLE tool for evaluating included studies. A study would receive a score of 1 if regarded as low risk in each domain. Based on the included studies, the scores ranged from 2 to 4. According to the evaluations of the studies, the results regarding sequence generation, random housing, allocation concealment, random outcome assessment, and blinding were all deemed unclear. No other sources of bias were detected for studies. The risk of bias assessment was impossible for one of the included studies since it was a poster (64). Detailed quality assessment results are presented in **Supplementary Materials 5** and **Figure 7**.

### 3.6 Publication bias

Funnel plots were created for the following outcomes: absolute testis weight, body weight, normal sperm morphology, seminiferous tubular diameter, serum testosterone level, sperm count, sperm motility, tissue GSH, MDA, and SOD. Evaluations for publication bias showed a significant small-study effect across the outcomes. Nevertheless, it is essential to interpret the results of the small-study effects tests with caution since they may be affected by other factors. For example, in the presence of between-study heterogeneity (the case of this study), the symmetry of funnel plots can be affected (30, 69). The funnel plots and Egger’s test results for small-effect studies are provided in the **Supplementary Material 6**.

## 4 Discussion

We demonstrated that melatonin could have beneficial effects against testicular abnormalities induced by radiotherapy and chemotherapy. Furthermore, we found that melatonin had a significantly greater impact on seminiferous tubular diameter, GPx, and FSH levels in preventive models rather than in therapeutic models. The strength of the melatonin’s effects on JTBS, sperm counts, and morphology also depended on the animal type. We also detected the model of intervention and rodent species as the sources of heterogeneity in different analyses.

### 4.1 Sperm quantity and quality

In the current meta-analysis, melatonin restored testicular injuries caused by radiotherapy and chemotherapy, which was indicated by increased spermatogonia and sperm count, normal morphology, motility, and viability, testis and epididymal weight, and seminiferous tubular height and diameter. These results agree with our previous meta-analyses, which revealed the beneficial impact of melatonin on testicular injuries induced by metabolic disorders, physical and toxic chemical triggers in animal models (70–72). Radio- and chemotherapy can cause disturbances in spermatogenesis through different mechanisms. These treatments may exert their effect by damaging DNA (DNA cross-link, breakage, alkylation, and intercalation) and induction of apoptosis, lipid peroxidation, increased oxidative stress, inflammation, hormonal imbalance, and mitochondrial damage, which result in abnormal sperm characteristics (6). Melatonin, as a potent antioxidant with anti-inflammatory and anti-apoptotic properties, can cross the cell membrane and penetrate the nucleus (73). As a direct free radical scavenger, melatonin can protect DNA against the destructive effects of Reactive oxygen species (ROS) induced by chemotherapy and radiotherapy (74). Melatonin’s ability to counteract the harmful effects of anti-cancer treatments can improve sperm morphology, motility, count, and viability. Zhang et al. reported that melatonin alleviates the cytotoxicity and anti-mitotic effects of busulfan, an alkylating chemotherapy agent, in the cultured spermatogonial progenitor cells. They found that the blockage of MT1 and MT2 in these cells antagonizes the observed effects of melatonin (32). In another *in-vitro* study, melatonin reversed the morphological changes caused by busulfan in the type A spermatogonial stem cells (19).

### 4.2 Reproductive hormone levels

Testosterone is produced by the Leydig cells located in the testis’s interstitial space, and the luteinizing hormone induces its secretion. Testosterone is required for normal spermatogenesis, and its serum concentration is positively associated with normal sperm morphology and higher live birth rates (75). Sertoli cells, located in the seminiferous tubules with critical roles in spermatogenesis and androgen synthesis, are also targeted by FSH (76). Melatonin administration elevated animal testosterone and reduced FSH levels in this meta-analysis. According to our recent meta-analysis, melatonin increases testosterone levels but does not affect FSH in rodents with toxin-induced testicular injuries (70). The existing body of research suggests that melatonin can inhibit the biosynthesis of FSH by decreasing the secretion of gonadotropin-releasing hormone (GnRH). Since melatonin administration has been observed to diminish the number of pituitary GnRH receptors, it is plausible that the observed reductions in plasma FSH concentrations may stem from inhibiting the pubertal increase in GnRH secretion (77).

Previous studies have yielded inconsistent findings regarding the impact of melatonin on testosterone levels (70–72). In this regard, da Costa et al. have reported that melatonin supplementation in pubescent rats may lead to a decline in testosterone levels in adulthood, potentially due to its influence on the estrogenic capacity of Leydig cells. Nonetheless, they also demonstrated that melatonin could exert a protective effect against the decrease in testosterone levels caused by the deleterious effects of diabetes, suggesting this protective effect may stem from melatonin’s ability to upregulate androgen receptor genes (78). Our results suggest melatonin’s protective effects against decreased testosterone levels induced by anti-cancer treatments. The blockages of MT1 and MT2 in the Leydig cell membrane downregulated steroidogenic genes (79). Melatonin can increase the expression of steroidogenic genes by binding to its nuclear receptors, including retinoic acid receptor-related orphan receptor α (RORα) (13). Furthermore, elevated melatonin levels improve testosterone synthesis by decreasing Leydig cells’ apoptosis (13), which may explain melatonin’s protective effect in our study. Nonetheless, there is contradictory evidence. Melatonin did not affect testosterone levels in animals with physical damage to the testes (71) and healthy human males (80, 81). Therefore, there is a need for more studies to determine melatonin’s effects on reproductive hormones and male infertility induced by oxidative stress.

### 4.3 Oxidative stress

Oxidative stress is among the causative factors for male infertility (82). In this regard, our results demonstrated melatonin’s beneficial effects on testicular enzymatic and non-enzymatic antioxidants in this study. By stimulating the activities of key antioxidant enzymes such as CAT, GSH-Px, SOD, and GSH while concurrently reducing the activity of MDA, a marker of lipid peroxidation, melatonin protects the testicular tissue against oxidative damage-induced radiation and chemotherapy. Previously, we detected similar efficacy of melatonin in metabolic disorders, physical- and chemical-induced testicular injuries (70–72). Furthermore, melatonin decreased microwave and radiofrequency electromagnetic radiation-induced oxidative stress (83). Literature suggests that melatonin increases antioxidant enzyme expression and activity during physiological and pathological conditions. These enzymes play a crucial role in mitigating the deleterious effects of free radicals by converting them into less reactive or non-toxic molecules, thus serving as a vital defense mechanism against oxidative stress. These enzymes can be recursively altered by free radicals, compromising their efficacy. In this context, melatonin acts as a potent scavenger of free radicals and can directly neutralize their destructive effects. Therefore, melatonin exerts a dual influence on the antioxidant system, both directly and indirectly, by regulating the activity of antioxidant enzymes and mitigating their damage by free radicals (84–86). In a recent study, Zhang et al. observed that the administration of cisplatin to mice results in apoptosis of Leydig cells by the downregulation of the SIRT1/Nrf2 signaling pathway, which plays a crucial role in anti-inflammatory response, anti-oxidative stress, and cell protection. However, the authors also suggest that melatonin can counteract the harmful effects of cisplatin by stimulating the SIRT1/Nrf2 pathway through its interaction with MT1/MT2 receptors (14, 87). Furthermore, melatonin, as a potent scavenger of reactive oxygen and nitrogen species, could also alleviate free radical formation by improving the electron transport chain efficiency of the inner mitochondrial membrane; by doing so, melatonin can reduce electron leakage, which is a significant source of free radical formation (88).

### 4.4 ER stress and apoptosis

In this study, we observed melatonin’s beneficial effects on reducing caspase-3 activity, which is a crucial mediator of apoptosis. This result aligns with our previous studies indicating melatonin protection against the apoptotic effects of metabolic disorders, physical injuries, environmental pollutants, and heavy metals on testes (70–72). Melatonin could alleviate testicular B-cell lymphoma-2 (Bcl-2)-associated X pro-apoptotic protein (BAX) and upregulate Bcl-2 anti-apoptotic protein following chemotherapy (20, 89). Radio- and chemotherapy could also trigger ER stress through different signaling pathways (including inositol-dependent protein 1 α (IRE1α), PRKR-like ER kinase (PERK)-eukaryotic translation initiating factor 2α (eIF2α), and MAPK), leading to cell death and potentially impairing male fertility (90).

Melatonin has been demonstrated to mitigate ER stress and inhibit intrinsic apoptotic pathways in anti-cancer treatment-induced ER stress (17, 19). In this regard, melatonin counteracted busulfan-induced ER stress and its downstream apoptotic proteins, including P53, caspases, and CCAAT enhancer binding protein (C/EBP) homologous protein (CHOP), in mouse testes and spermatogonial stem cells (19). Melatonin may reverse radiotherapy and chemotherapy-induced ER stress by suppressing the phosphatidylinositol 3-kinase (PI3K)/protein kinase B (AKT) pathway (17). Eliminating ER stress by melatonin could improve blood-testis barrier impairment and, thereby, spermatogenesis abnormalities following busulfan treatment (91).

### 4.5 Inflammation

Pro-inflammatory cytokines play a key role in maintaining the normal physiological functions of testicular cells by acting as growth and differentiation factors (92). However, their increased levels during acute and chronic genitourinary tract inflammation are linked to oxidative stress and male infertility (93). Melatonin supplementation is reported to reduce testicular inflammation in infertile men (94). It may also reverse the radiotherapy- and chemotherapy-induced male reproductive toxicities by attenuating the testicular levels of inflammatory cytokines, including interleukin-1β (IL-1β), tumor necrosis factor-α (TNF-α), and interleukin-6 (IL-6) (2, 5, 17, 20, 62, 95). These effects may be attributed to melatonin’s inhibition of the p38 mitogen-activated protein kinase (MAPK) signaling pathway and, subsequently, toll-like receptor 4 (TLR-4) and nuclear factor kappa B (NF-κB) in the testes (96). The activated TLRs are associated with low sperm motility, sperm apoptosis, and male infertility (97). Yet, more studies should be performed to evaluate other affected cytokines and cascades by exogenous melatonin.

### 4.6 Limitations

Our study had several limitations. First, our data was extracted from animal studies, and it is unclear whether such effects could be translated to humans. Furthermore, most available animal studies evaluating the effects of melatonin therapy on male infertility used rodent models, making the conclusions hard to generalize to other animals. Second, there was high methodological and statistical heterogeneity between the included studies. Third, our meta-analysis is also limited by the low quality of the eligible studies and a high level of publication bias. Also, a dose-response meta-analysis was not feasible due to insufficient data and differences in the route of administration. Finally, none of the included studies have reported and evaluated possible adverse outcomes.

## 5 Conclusion

In the current meta-analysis of animal studies, we conclude melatonin’s protective influence on the side effects of radiotherapy and chemotherapy on testicular tissue. Improving testicular function and morphology, ameliorating hormone levels, and alleviating oxidative stress and apoptosis are some proposed mechanisms for the observed effects of melatonin. However, more meticulous animal studies should be performed to clarify other potential underlying mechanisms. Future studies are recommended to evaluate melatonin dose responses to provide doses with anti-infertility effects.

## Data availability statement

The original contributions presented in the study are included in the article/**Supplementary Material**. Further inquiries can be directed to the corresponding author.

## Author contributions

The study was conceptualized by NA and ND and designed by AS, ND, SS-Z, and ARS. ND, AS, and ET searched the databases. ZE, ZR, ND, and MS extracted the data. MS and ND performed the quality assessment. AS performed meta-analyses. AS visualized the data and designed the graphical abstract. Drafts of the manuscript were provided by ND, AS, and SS-Z. NA, ARS, ND, and ET supervised the study. All authors made substantial contributions to the article and endorsed the final version.

## References

[1] Sung H Ferlay J Siegel RL Laversanne M Soerjomataram I Jemal A . Global cancer statistics 2020: GLOBOCAN estimates of incidence and mortality worldwide for 36 cancers in 185 countries. CA: Cancer J Clin (2021) 71(3):209–49. doi: 10.3322/caac.21660 33538338

[2] Ghafouri-Fard S Shoorei H Abak A Seify M Mohaqiq M Keshmir F . Effects of chemotherapeutic agents on male germ cells and possible ameliorating impact of antioxidants. Biomedicine Pharmacother. (2021) 142:112040. doi: 10.1016/j.biopha.2021.112040 34416630

[3] Roychoudhury S Das A Panner Selvam MK Chakraborty S Slama P Sikka SC . Recent publication trends in radiotherapy and male infertility over two decades: a scientometric analysis. Front Cell Dev Biol (2022) 889. doi: 10.3389/fcell.2022.877079 PMC9133602 35646894

[4] Cetinkaya-Un B Un B Akpolat M Andic F Yazir Y . Human amnion membrane-derived mesenchymal stem cells and conditioned medium can ameliorate X-irradiation-induced testicular injury by reducing endoplasmic reticulum stress and apoptosis. Reprod Sci (2022) 29(3):1–11. doi: 10.1007/s43032-021-00753-6 34642916

[5] Qu N Itoh M Sakabe K . Effects of chemotherapy and radiotherapy on spermatogenesis: The role of testicular immunology. Int J Mol Sci (2019) 20(4):957. doi: 10.3390/ijms20040957 30813253 PMC6413003

[6] Meistrich ML . Effects of chemotherapy and radiotherapy on spermatogenesis in humans. Fertility sterility. (2013) 100(5):1180–6. doi: 10.1016/j.fertnstert.2013.08.010 PMC3826884 24012199

[7] Brydøy M Fosså SD Dahl O Bjøro T . Gonadal dysfunction and fertility problems in cancer survivors. Acta Oncologica (2007) 46(4):480–9. doi: 10.1080/02841860601166958 17497315

[8] Okada K Fujisawa M . Recovery of spermatogenesis following cancer treatment with cytotoxic chemotherapy and radiotherapy. World J men's Health (2019) 37(2):166–74. doi: 10.5534/wjmh.180043 PMC6479085 30588779

[9] Farhood B Mortezaee K Haghi-Aminjan H Khanlarkhani N Salehi E Nashtaei MS . A systematic review of radiation-induced testicular toxicities following radiotherapy for prostate cancer. J Cell Physiol (2019) 234(9):14828–37. doi: 10.1002/jcp.28283 30740683

[10] Lai JC Tandon P Bernal W Tapper EB Ekong U Dasarathy S . Malnutrition, frailty, and sarcopenia in patients with cirrhosis: 2021 practice guidance by the american association for the study of liver diseases. Hepatol (Baltimore Md) (2021) 74(3):1611–44. doi: 10.1002/hep.32049 PMC9134787 34233031

[11] Kozioł K Broda D Romerowicz-Misielak M Nowak S Koziorowski M . Melatonin concentration in peripheral blood and melatonin receptors (MT1 and MT2) in the testis and epididymis of male roe deer during active spermatogenesis. Theriogenology (2020) 149:25–37. doi: 10.1016/j.theriogenology.2020.03.025 32234648

[12] Lampiao F Du Plessis SS . New developments of the effect of melatonin on reproduction. World J Obstetrics Gynecol (2013) 2(2):8–15. doi: 10.5317/wjog.v2.i2.8

[13] Yang M Guan S Tao J Zhu K Lv D Wang J . Melatonin promotes male reproductive performance and increases testosterone synthesis in mamMalian Leydig cells. Biol Reprod (2021) 104(6):1322–36. doi: 10.1093/biolre/ioab046 33709108

[14] Zhang J Fang Y Tang D Xu X Zhu X Wu S . Activation of MT1/MT2 to protect testes and leydig cells against cisplatin-induced oxidative stress through the SIRT1/Nrf2 signaling pathway. Cells. (2022) 11(10):1690. doi: 10.3390/cells11101690 35626727 PMC9139217

[15] Li W Kwok CC-H Chan DC-W Ho AW-Y Ho C-S Zhang J . Disruption of sleep, sleep-wake activity rhythm, and nocturnal melatonin production in breast cancer patients undergoing adjuvant chemotherapy: prospective cohort study. Sleep Med (2019) 55:14–21. doi: 10.1016/j.sleep.2018.11.022 30743205

[16] de Castro TB Bordin-Junior NA de Almeida EA de Campos Zuccari DAP . Evaluation of melatonin and AFMK levels in women with breast cancer. Endocrine. (2018) 62:242–9. doi: 10.1007/s12020-018-1624-2 29797213

[17] Ma Z Xu L Liu D Zhang X Di S Li W . Utilizing melatonin to alleviate side effects of chemotherapy: a potentially good partner for treating cancer with ageing. Oxid Med Cell Longevity (2020) 2020. doi: 10.1155/2020/6841581 PMC7260648 32566095

[18] Najafi M Shirazi A Motevaseli E Geraily G Norouzi F Heidari M . The melatonin immunomodulatory actions in radiotherapy. Biophys Rev (2017) 9:139–48. doi: 10.1007/s12551-017-0256-8 PMC5425818 28510090

[19] Cui Y Ren L Li B Fang J Zhai Y He X . Melatonin relieves busulfan-induced spermaTogonial stem cell apoptosis of mouse testis by inhibiting endoplasmic reticulum stress. Cell Physiol Biochem (2017) 44(6):2407–21. doi: 10.1159/000486165 29268276

[20] Moradi M Goodarzi N Faramarzi A Cheraghi H Hashemian AH Jalili C . Melatonin protects rats testes against bleomycin, etoposide, and cisplatin-induced toxicity via mitigating nitro-oxidative stress and apoptosis. Biomedicine Pharmacotherapy. (2021) 138:111481. doi: 10.1016/j.biopha.2021.111481 33752059

[21] Take G Erdogan D Helvacioglu F Göktas G Ozbey G Uluoglu C . Effect of melatonin and time of administration on irradiation-induced damage to rat testes. Braz J Med Biol Res (2009) 42(7):621–8. doi: 10.1590/S0100-879X2009000700006 19578641

[22] Madhu P Reddy KP Reddy PS . Role of melatonin in mitigating chemotherapy-induced testicular dysfunction in Wistar rats. Drug Chem Toxicol (2016) 39(2):137–46. doi: 10.3109/01480545.2015.1055359 26072956

[23] Haghi-Aminjan H Asghari MH Farhood B Rahimifard M Hashemi Goradel N Abdollahi M . The role of melatonin on chemotherapy-induced reproductive toxicity. J Pharm Pharmacol (2018) 70(3):291–306. doi: 10.1111/jphp.12855 29168173

[24] Page MJ McKenzie JE Bossuyt PM Boutron I Hoffmann TC Mulrow CD . The PRISMA 2020 statement: an updated guideline for reporting systematic reviews. BMJ (2021) 372:n71. doi: 10.1136/bmj.n71 33782057 PMC8005924

[25] Ouzzani M Hammady H Fedorowicz Z Elmagarmid A . Rayyan—a web and mobile app for systematic reviews. Systematic Rev (2016) 5(1):210. doi: 10.1186/s13643-016-0384-4 PMC5139140 27919275

[26] Hooijmans CR Rovers MM de Vries RB Leenaars M Ritskes-Hoitinga M Langendam MW . SYRCLE's risk of bias tool for animal studies. BMC Med Res methodology. (2014) 14:43. doi: 10.1186/1471-2288-14-43 PMC4230647 24667063

[27] Abbasnezhad A Choghakhori R Kashkooli S Alipour M Asbaghi O Mohammadi R . Effect of L-carnitine on liver enzymes and biochemical factors in hepatic encephalopathy: A systematic review and meta-analysis. J Gastroenterol Hepatol. (2019) 34(12):2062–70. doi: 10.1111/jgh.14765 31254469

[28] Luo D Wan X Liu J Tong T . Optimally estimating the sample mean from the sample size, median, mid-range, and/or mid-quartile range. Stat Methods Med Res (2018) 27(6):1785–805. doi: 10.1177/0962280216669183 27683581

[29] Shi J Luo D Weng H Zeng XT Lin L Chu H . Optimally estimating the sample standard deviation from the five-number summary. Res Synth Methods (2020) 11(5):641–54. doi: 10.1002/jrsm.1429 32562361

[30] Egger M Davey Smith G Schneider M Minder C . Bias in meta-analysis detected by a simple, graphical test. Bmj (1997) 315(7109):629–34. doi: 10.1136/bmj.315.7109.629 PMC2127453 9310563

[31] Simsek A Otunctemur A Özcan L Cilli M Polat E Somay A . Preventive effects of melatonin in cisplatin and cyclophosphamide associated testes damage. Eur Urol Suppl (2008) 7:93–. doi: 10.1016/S1569-9056(08)60093-7

[32] Zhang X Xia Q Wei R Song H Mi J Lin Z . Melatonin protects spermaTogonia from the stress of chemotherapy and oxidation via eliminating reactive oxidative species. Free Radic Biol Med (2019) 137:74–86. doi: 10.1016/j.freeradbiomed.2019.04.009 30986493

[33] Abou-El-Naga A-M Mousa S-A Althobaiti F Fayad E Fahim E-S . Ameliorative effects of melatonin and zinc oxide nanoparticles treatment against adverse effects of busulfan induced infertility in male albino mice. BIOCELL (2022) 46(2):535–45. doi: 10.32604/biocell.2022.017739

[34] Abd El Aziz DH Metwally HG . The effect of stem cell therapy versus melatonin on the changes induced by busulfan in the testes of adult rat: histological and immunohistochemical studies. Egyptian J Histol (2013) 36(1):175–84. doi: 10.1097/01.EHX.0000425579.77855.ea

[35] Aboelwafa HR Ramadan RA El-Kott AF Abdelhamid FM . The protective effect of melatonin supplementation against taxol-induced testicular cytotoxicity in adult rats. Braz J Med Biol Res (2022) 55:e11614. doi: 10.1590/1414-431x2021e11614 35137851 PMC8851920

[36] Alp BF Kesik V Malkoç E Yiğit N Saldır M Babacan O . The effect of melatonin on procarbazine induced testicular toxicity on rats. Syst Biol Reprod Med (2014) 60(6):323–8. doi: 10.3109/19396368.2014.930212 25140409

[37] Ateşşahin A Sahna E Türk G Ceribaşi AO Yilmaz S Yüce A . Chemoprotective effect of melatonin against cisplatin-induced testicular toxicity in rats. J Pineal Res (2006) 41(1):21–7. doi: 10.1111/j.1600-079X.2006.00327.x 16842537

[38] Baş E Nazıroğlu M . Treatment with melatonin and selenium attenuates docetaxel-induced apoptosis and oxidative injury in kidney and testes of mice. Andrologia. (2019) 51(8):e13320. doi: 10.1111/and.13320 31131920

[39] Cebi Sen C Yumusak N Atilgan HI Sadic M Koca G Korkmaz M . The protective effect of melatonin on sperm quality in rat after radioiodine treatment. Andrologia. (2018) 50(4):e12962. doi: 10.1111/and.12962 29430687

[40] Chabra A Shokrzadeh M Naghshvar F Salehi F Ahmadi A . Melatonin ameliorates oxidative stress and reproductive toxicity induced by cyclophosphamide in male mice. Hum Exp Toxicol (2014) 33(2):185–95. doi: 10.1177/0960327113489052 23703819

[41] Edrees Z kader H Embaby A hameed E . The effect of melatonin on the testes of rats treated with cyclophosphamide: Histological and immunohistochemical study. Egyptian J Histol (2012) 35:822–32. doi: 10.1097/01.EHX.0000419785.84206.d2

[42] El-Shafaei A Abdelmaksoud R Elshorbagy A Zahran N Elabd R . Protective effect of melatonin versus montelukast in cisplatin-induced seminiferous tubule damage in rats. Andrologia. (2018) 50(9):e13077. doi: 10.1111/and.13077 30019386

[43] Eren H Mercantepe T Tumkaya L Mercantepe F Dil E Horsanali MO . Evaluation of the protective effects of amifostine and melatonin against cisplatin induced testis injury via oxidative stress and apoptosis in rats. Exp Mol Pathol (2020) 112:104324. doi: 10.1016/j.yexmp.2019.104324 31697930

[44] Filobbos S Amin N Yacoub M Abd El_Hakim KR . Possible protective effect of melatonin on cisplatin-induced testicular toxicity in adult albino rats. A histological and immunohistochemical study. Egyptian J Histol (2020) 43(3):891–901. doi: 10.21608/EJH.2020.21561.1221

[45] Ghasemi FM Faghani M Khajehjahromi S Bahadori M Nasiri EE Hemadi M . Effect of melatonin on proliferative activity and apoptosis in spermatogenic cells in mouse under chemotherapy. J Reprod Contraception (2010) 21(2):79–94. doi: 10.1016/S1001-7844(10)60016-8

[46] Hussein MR Abu-Dief EE Abou El-Ghait AT Adly MA Abdelraheem MH . Melatonin and roentgen irradiation of the testis. Fertil Steril. (2006) 86(3):750–2. doi: 10.1016/j.fertnstert.2006.02.094 16854416

[47] Ilbey YO Ozbek E Simsek A Otunctemur A Cekmen M Somay A . Potential chemoprotective effect of melatonin in cyclophosphamide- and cisplatin-induced testicular damage in rats. Fertil Steril (2009) 92(3):1124–32. doi: 10.1016/j.fertnstert.2008.07.1758 18829000

[48] Kamal El-Dein EMKE-D Anees LM . Ameliorative role of melatonin against cypermethrin or gamma irradiation induced testicular damage in male rats. Int J Radiat Res (2020) 18(4):765–76. doi: 10.18869/acadpub.ijrr.18.4.765

[49] Khan S Adhikari JS Rizvi MA Chaudhury NK . Radioprotective potential of melatonin against 60Co γ-ray-induced testicular injury in male C57BL/6 mice. J Biomed Science. (2015) 22(1):61. doi: 10.1186/s12929-015-0156-9 PMC4514449 26205951

[50] Kushwaha R Nishad DK Bhatnagar A Khar RK . Melatonin-caffeine combination modulates gamma radiation-induced sperm malformations in C57BL/6 male mice at sublethal dose of gamma radiation. J Pharm Bioallied Sci (2021) 13(2):268–75. doi: 10.4103/jpbs.JPBS_303_20 PMC8291117 34349489

[51] Lee K-M Lee I-C Kim S-H Moon C Park S-H Shin D-H . Melatonin attenuates doxorubicin-induced testicular toxicity in rats. Andrologia (2012) 44(s1):796–803. doi: 10.1111/j.1439-0272.2011.01269.x 22212014

[52] Manda K Bhatia AL . Prophylactic action of melatonin against cyclophosphamide-induced oxidative stress in mice. Cell Biol Toxicol (2003) 19(6):367–72. doi: 10.1023/B:CBTO.0000013342.17370.16 15015761

[53] Mirhoseini M Saki G Hemadi M Khodadadi A Mohammadi Asl J . Melatonin and testicular damage in busulfan treated mice. Iran Red Crescent Med J (2014) 16(2):e14463. doi: 10.5812/ircmj.14463 24719743 PMC3965876

[54] Moghadam MT Dadfar R Khorsandi L . The effects of ozone and melatonin on busulfan-induced testicular damage in mice. JBRA Assist Reprod (2021) 25(2):176–84. doi: 10.5935/1518-0557.20200081 PMC8083863 33507719

[55] Mohammad Ghasemi F FaghaniLangroudi M Falah Karkan M . The protective effect of melatonin on sperm parameters, epididymis and seminal vesicle morphology in adult mouse treated with busulfan. Anatomical Sci J (2010) 8(30).

[56] Olayaki LA Adeyemi WJ Adeyemi E Osawaru O Busura I Jimoh S . Melatonin enhanced the restoration of biochemical profile in chlorambucil treated-rats: examination of after-withdrawal effects of the drug. J Afr Assoc Physiol Sci (2020) 7(2):80–7.

[57] Patil L Balaraman R . Effect of melatonin on doxorubicin induced testicular damage in rats. Int J PharmTech Res CODEN( USA): IJPRIF ISSN. (2023) 1:974–4304.

[58] Sukhorum W Umka Welbat J Krutsri S Iamsaard Comma S . Protective effect of melatonin against methotrexate-induced testicular damage in the rat model: An experimental study. Int J Reprod Biomed (2020) 18(5):327–38. doi: 10.18502/ijrm.v13i5.7153 PMC7306061 32637861

[59] Tajabadi E Javadi A Azar NA Najafi M Shirazi A Shabeeb D . Radioprotective effect of a combination of melatonin and metformin on mice spermatogenesis: A histological study. Int J Reprod Biomed (2020) 18(12):1073–80. doi: 10.18502/ijrm.v18i12.8029 PMC7778753 33426418

[60] Tawfik SS Mansour HH El-Shamy E Sallam MH . Radioprotective effect and follow-up of melatonin as antifertility drug in male adult mice submitted to whole-body γ Irradiation. Egyptian J Radiat Sci App (2006) 19(2):331–51.

[61] Torabi F Malekzadeh Shafaroudi M Rezaei N . Combined protective effect of zinc oxide nanoparticles and melatonin on cyclophosphamide-induced toxicity in testicular histology and sperm parameters in adult Wistar rats. Int J Reprod Biomed (2017) 15(7):403–12. doi: 10.29252/ijrm.15.7.403 PMC5601931 29202124

[62] Wang Y Zhao TT Zhao HY Wang H . Melatonin protects methotrexate-induced testicular injury in rats. Eur Rev Med Pharmacol Sci (2018) 22(21):7517–25. doi: 10.26355/eurrev_201811_16293 30468501

[63] Wang Z Teng Z Wang Z Song Z Zhu P Li N . Melatonin ameliorates paclitaxel-induced mice spermatogenesis and fertility defects. J Cell Mol Med (2022) 26(4):1219–28. doi: 10.1111/jcmm.17177 PMC8831955 35001532

[64] Yalçınkaya F Gökçe A Guven O Davarcı M Cikim G Yekeler H . N88 Protective effect of vitamine E and melatonin against radiation induced damage in testis of rat. Eur Urol Suppl (2009) 8:599–. doi: 10.1016/S1569-9056(09)74862-6

[65] Zangoie R Eshraghi H Shirian S Kadivar A Nazari H Aali E . Melatonin synergistically enhances protective effect of atorvastatin against busulfan-induced spermatogenesis injuries in a rat model. Comp Clin Pathol (2020) 29(1):161–6. doi: 10.1007/s00580-019-03040-8

[66] Zi T Liu Y Zhang Y Wang Z Wang Z Zhan S . Protective effect of melatonin on alleviating early oxidative stress induced by DOX in mice spermatogenesis and sperm quality maintaining. Reprod Biol Endocrinol (2022) 20(1):105. doi: 10.1186/s12958-022-00977-4 35850689 PMC9290234

[67] Mohamadghasemi F Faghani M Khajehjahromi S . The protective effects of melatonin on the histological changes of testis in busulfan-treated adult mice. J Reprod Infertil (2010) 11(2):67.

[68] Ferdosi Khosroshahi A Bakhtiari M Soleimani Rad J Koroji M Roshangar L Janzadeh A . Study of the effect of exogenous melatonin on sperm fertility in busulfan induced oligospermic of pinealectomeized rat. Razi J Med Sci (2013) 20(110):77–86.

[69] Sterne JAC Egger M . Regression methods to detect publication and other bias in meta-analysis. Publ Bias Meta-Analysis (2005) 99–110. doi: 10.1002/0470870168.ch6

[70] Dehdari Ebrahimi N Parsa S Nozari F Shahlaee MA Maktabi A Sayadi M . Protective effects of melatonin against the toxic effects of environmental pollutants and heavy metals on testicular tissue: A systematic review and meta-analysis of animal studies. Front Endocrinol (2023) 14. doi: 10.3389/fendo.2023.1119553 PMC9922902 36793277

[71] Dehdari Ebrahimi N Shojaei-Zarghani S Taherifard E Dastghaib S Parsa S Mohammadi N . Protective effects of melatonin against physical injuries to testicular tissue: A systematic review and meta-analysis of animal models. Front Endocrinol (2023) 14. doi: 10.3389/fendo.2023.1119553 PMC9927015 36798664

[72] Dehdari Ebrahimi N Sadeghi A Ala M Ebrahimi F Pakbaz S Azarpira N . Protective effects of melatonin against oxidative stress induced by metabolic disorders in the male reproductive system: A systematic review and meta-analysis of rodent models. Front Endocrinol (2023) 14. doi: 10.3389/fendo.2023.1202560 PMC10354453 37476491

[73] Reiter RJ Tan DX Mayo JC Sainz RM Leon J Czarnocki Z . Melatonin as an antioxidant: biochemical mechanisms and pathophysiological implications in humans. Acta Biochim Polonica. (2003) 50(4):1129–46. doi: 10.18388/abp.2003_3637 14740000

[74] Ressmeyer AR Mayo JC Zelosko V Sáinz RM Tan DX Poeggeler B . Antioxidant properties of the melatonin metabolite N1-acetyl-5-methoxykynuramine (AMK): scavenging of free radicals and prevention of protein destruction. Redox Rep Commun Free Radical Res (2003) 8(4):205–13. doi: 10.1179/135100003225002709 14599344

[75] Trussell J Coward RM Santoro N Stetter C Kunselman A Diamond MP . Association between testosterone, semen parameters, and live birth in men with unexplained infertility in an intrauterine insemination population. Fertility sterility. (2019) 111(6):1129–34. doi: 10.1016/j.fertnstert.2019.01.034 PMC6557574 30982604

[76] Oduwole OO Peltoketo H Huhtaniemi IT . Role of follicle-stimulating hormone in spermatogenesis. Front endocrinology. (2018) 9:763. doi: 10.3389/fendo.2018.00763 PMC6302021 30619093

[77] Lang U Aubert ML Rivest RW Vinas-Bradtke JC Sizonenko PC . Daily afternoon administration of melatonin does not irreversibly inhibit sexual maturation in the male rat. Endocrinology (1984) 115(6):2303–10. doi: 10.1210/endo-115-6-2303 6499770

[78] da Costa CF Gobbo MG Taboga SR Pinto-Fochi ME Góes RM . Melatonin intake since weaning ameliorates steroidogenic function and sperm motility of streptozotocin-induced diabetic rats. Andrology (2016) 4(3):526–41. doi: 10.1111/andr.12158 27037637

[79] Gao Y Wu X Zhao S Zhang Y Ma H Yang Z . Melatonin receptor depletion suppressed hCG-induced testosterone expression in mouse Leydig cells. Cell Mol Biol letters. (2019) 24(1):1–14. doi: 10.1186/s11658-019-0147-z PMC6416941 30915128

[80] Luboshitzky R Levi M Shen-Orr Z Blumenfeld Z Herer P Lavie P . Long-term melatonin administration does not alter pituitary-gonadal hormone secretion in normal men. Hum reproduction. (2000) 15(1):60–5. doi: 10.1093/humrep/15.1.60 10611189

[81] Zizzo J Reddy R Kulkarni N Blachman-Braun R Ramasamy R . Impact of low-dose melatonin supplementation on testosterone levels in US adult males. Urology (2022) 169:92–5. doi: 10.1016/j.urology.2022.07.048 35963395

[82] Mannucci A Argento FR Fini E Coccia ME Taddei N Becatti M . The impact of oxidative stress in male infertility. Front Mol Biosci (2022) 8:1344. doi: 10.3389/fmolb.2021.799294 PMC8766739 35071326

[83] Shokri M Shamsaei ME Malekshah AK Amiri FT . The protective effect of melatonin on radiofrequency electromagnetic fields of mobile phone-induced testicular damage in an experimental mouse model. Andrologia (2020) 52(11):e13834. doi: 10.1111/and.13834 33040351

[84] Aysun H Burcu B . (2018), Ch. 3.

[85] Chainy GBN Sahoo DK . Hormones and oxidative stress: an overview. Free Radical Res (2020) 54(1):1–26. doi: 10.1080/10715762.2019.1702656 31868060

[86] Sahoo DK Chainy GBN . Hormone-linked redox status and its modulation by antioxidants. Vitam Horm. (2023) 121:197–246. doi: 10.1016/bs.vh.2022.10.007 36707135

[87] Chung J-Y Chen H Zirkin B . Sirt1 and Nrf2: regulation of Leydig cell oxidant/antioxidant intracellular environment and steroid formation. Biol Reprod (2021) 105(5):1307–16. doi: 10.1093/biolre/ioab150 PMC8598996 34363387

[88] Leon J Acuña-Castroviejo D Sainz RM Mayo JC Tan DX Reiter RJ . Melatonin and mitochondrial function. Life Sci (2004) 75(7):765–90. doi: 10.1016/j.lfs.2004.03.003 15183071

[89] Zi T Liu Y Zhang Y Wang Z Wang Z Zhan S . Protective effect of melatonin on alleviating early oxidative stress induced by DOX in mice spermatogenesis and sperm quality maintaining. Reprod Biol Endocrinol (2022) 20(1):1–11. doi: 10.1186/s12958-022-00977-4 35850689 PMC9290234

[90] Karna KK Shin YS Choi BR Kim HK Park JK . The role of endoplasmic reticulum stress response in male reproductive physiology and pathology: A review. World J Mens Health (2020) 38(4):484–94. doi: 10.5534/wjmh.190038 PMC7502313 31385474

[91] Zhao J Wang M Wang Y Xu J Ma C Tang Y . Endoplasmic reticulum stress promotes blood-testis barrier impairment in mice with busulfan-induced oligospermia through PERK-eIF2α signaling pathway. Toxicology (2022) 473:153193. doi: 10.1016/j.tox.2022.153193 35533795

[92] Hales DB Diemer T Hales KH . Role of cytokines in testicular function. Endocrine. (1999) 10:201–17. doi: 10.1007/BF02738619 10484284

[93] Azenabor A Ekun AO Akinloye O . Impact of inflammation on male reproductive tract. J Reprod Infertil (2015) 16(3):123. 26913230 PMC4508350

[94] Riviere E Rossi SP Tavalieri YE de Toro MMM Ponzio R Puigdomenech E . Melatonin daily oral supplementation attenuates inflammation and oxidative stress in testes of men with altered spermatogenesis of unknown aetiology. Mol Cell Endocrinology (2020) 515:110889. doi: 10.1016/j.mce.2020.110889 32622722

[95] Akaras N Bal T Atilay H Selli J Halici M . Protective effects of agomelatine on testicular damage caused by bortezomib. Biotech Histochem (2017) 92(8):552–9. doi: 10.1080/10520295.2017.1350748 29161153

[96] Deng S-L Zhang B-L Reiter RJ Liu Y-X . Melatonin ameliorates inflammation and oxidative stress by suppressing the p38MAPK signaling pathway in LPS-induced sheep orchitis. Antioxidants (2020) 9(12):1277. doi: 10.3390/antiox9121277 33327643 PMC7765110

[97] Fujita Y Mihara T Okazaki T Shitanaka M Kushino R Ikeda C . Toll-like receptors (TLR) 2 and 4 on human sperm recognize bacterial endotoxins and mediate apoptosis. Hum Reprod (2011) 26(10):2799–806. doi: 10.1093/humrep/der234 PMC3174031 21775336

