# Supplementary material for: Protective effects of exogenous melatonin therapy against oxidative stress to male reproductive tissue caused by anti-cancer chemical and radiation therapy: a systematic review and meta-analysis of animal studies
Source: Front Endocrinol (Lausanne). 2023 Aug 28;14:1184745. doi: 10.3389/fendo.2023.1184745 (PMC10494246; doi:10.3389/fendo.2023.1184745)
Supplement: Supplementary file 1 [file DataSheet_1.zip › Supplementary Material/Supplementary Material 2.DOCX]

| First author [year] | Type of OS | Administration route of OS | Duration of exposure to OS | Cumulative dose of OS | Duration of melatonin therapy | Cumulative dose of melatonin therapy | Administration route of melatonin |
| --- | --- | --- | --- | --- | --- | --- | --- |
| Wang [2018] (1) | Chemical agent (Methotrexate) | Oral | Single dose | 150 mg/kg | 28 days | 560 mg/kg | Oral |
| Wang [2022] (2) | Chemical agent (Paclitaxel) | IP | Single dose | 10 mg/kg | 14 days | 140 mg/kg | IP |
| Yalcınkaya [2009] (3) | Radiation (Gamma radiation) | Radiation | Single dose | 360 cGy | 1 day | 100 mg/kg | IP |
| Zangoie [2019] (4) | Chemical agent (Busulfan) | IP | 2 doses | 35 mg/kg | 28 days | 28 mg/kg | IP |
| Zhang [2022] (5) | Chemical agent (Cisplatin) | IP | 4, 5, 7, 9, 10, and 12 days | 12, 13, 15, 17, 18, and 20 mg/kg | 21 days | 210 mg/kg | IP |
| Zi [2022] (6) | Chemical agent (Doxorubicin) | IP | Single dose | 10 mg/kg | 35 days | 350 mg/kg | IP |
| Hussein [2006] (7) | Radiation (Roentgen radiation) | Radiation | Single dose | 8 Gy | 1 day | 100 mg/kg | IP |
| Khan [2015] (8) | Radiation (Gamma radiation) | Radiation | 5 minutes | 5 Gy | 1 day | 100 mg/kg | IP |
| Kushwaha [2021] (9) | Radiation (Gamma radiation) | Radiation | Single dose | 2 Gy | 1 day | 100 mg/kg | IP |
| Lee [2012] (10) | Chemical agent (Doxorubicin) | IP | Single dose | 10 mg/kg | 10 days | 150 mg/kg | Oral |
| Madhu [2015] (11) | Chemical agent (Cisplatin + Vinblastine + Bleomycin) | IP | 63 days | Cisplatin 9 mg/kg + Vinblastine 0.9 mg/kg + Bleomycin 4.5 mg/kg | 63 days | 90 mg/kg | IP |
| Manda [2003] (12) | Chemical agent (Cyclophosphamide) | IP | Single dose | 75 mg/kg | 15 days | 1.5 mg/kg | Oral |
| Mirhoseini [2014] (13) | Chemical agent (Busulfan) | IP | Single dose | 40 mg/kg | 60 days | 1200 mg/kg | IP |
| Taheri Moghadam [2021] (14) | Chemical agent (Busulfan) | IP | Single dose | 30 mg/kg | 7 days | 70 mg/kg | IP |
| Moradi [2021] (15) | Chemical agent (Bleomycin + Etoposide + Cisplatin) | IP | Single dose | Multiple drugs | 21 days | 210 and 420 mg/kg | IP |
| Cebi Sen [2018] (16) | Radiation (Radioactive iodine) | Oral | Single dose | 111 MBq | 10 days | 120 mg/kg | IP |
| Patil [2009] (17) | Chemical agent (Doxorubicin) | IP | 5 days | 15 mg/kg | 28 days | 168 mg/kg | Oral |
| Aboelwafa [2022] (18) | Chemical agent (Taxol) | IP | 5 days | 37.5 mg/kg | 30 days | 300 mg/kg | IP |
| Alp [2014] (19) | Chemical agent (Procarbazine) | Oral | 4 days | 250 mg/kg | 20 days | 200 mg/kg | IP |
| Atessahin [2006] (20) | Chemical agent (Cisplatin) | IP | Single dose | 7 mg/kg | 5 days | 50 mg/kg | IP |
| Baş [2019] (21) | Chemical agent (Docetaxel) | IP | Single dose | 30 mg/kg | 7 days | 70 mg/kg | IP |
| Chabra [2014] (22) | Chemical agent (Cisplatin) | IP | Single dose | 200 mg/kg | 5 days | 12.5, 25, 50, and 100 mg/kg | IP |
| Cui [2017] (23) | Chemical agent (Busulfan) | N/A | Single dose | 30 mg/kg | 28 days | 280 mg/kg | N/A |
| Edrees [2012] (24) | Chemical agent (Cisplatin) | IP | 2 days | 200 mg/kg | 14 and 35 days | 140 and 350 mg/kg | IP |
| Kamal El-Dein [2020] (25) | Radiation (Gamma radiation) | Radiation | 8 days | 8 Gy | 29 days | 290 mg/kg | Oral |
| El-shafaei [2018] (26) | Chemical agent (Cisplatin) | IP | Single dose | 7 mg/kg | 10 days | 200 mg/kg | Oral |
| Yilmaz [2019] (27) | Chemical agent (Cisplatin) | IP | Single dose | 7.5 mg/kg | 5 days | 25 mg/kg | IP |
| Filobbos [2020] (28) | Chemical agent (Cisplatin) | IP | Single dose | 7 mg/kg | 15 days | 120 mg/kg | Oral |
| Mohamad Ghasemi [2010] (i) (29) | Chemical agent (Busulfan) | IP | Single dose | 20 mg/kg | 5 days | 50 mg/kg | IP |
| Ilbey [2008] (30) | Chemical agent (Cisplatin) | IP | 5 days | 35 mg/kg | 1 day | 10 mg/kg | IP |
|  | Chemical agent (Cyclophosphamide) | Oral | Single dose | 100 mg/kg |  |  |  |
| Mohamad Ghasemi [2010] (ii) (31) | Chemical agent (Busulfan) | IP | Single dose | 20 mg/kg | 5 days | 50 mg/kg | IP |
| Ferdosi Khosroshahi [2013] (Farsi) (32) | Chemical agent (Busulfan) | IP | Single dose | 20 mg/kg | 50 days | 25 mg | IP |
| Mohammd Ghasemi [2009] (Farsi) (33) | Chemical agent (Busulfan) | IP | Single dose | 20 mg/kg | 5 days | 50 mg | IP |
| Olayaki [2019] (34) | Chemical agent (Chlorambucil) | Oral | 21 days | 4.2 mg/kg | 21 days | 210 mg/kg | Oral |
| Tawfik [2006] (35) | Radiation (Gamma radiation) | Radiation | N/A | 2 Gy | 20 days | 200 mg/kg | IP |
| Sukhorum [2020] (36) | Chemical agent (Methotrexate) | IV | 15 days | 1125 mg/kg | 15 and 30 days | 120 and 240 mg/kg | IP |
| Tajabadi [2020] (37) | Radiation (Gamma radiation) | Radiation | Single dose | 2 Gy | 1 day | 100 mg/kg | N/A |
| Torabi [2017] (38) | Chemical agent (Cyclophosphamide) | IP | 8 weeks | 480 mg/kg | 56 days | 80 mg/kg | IP |
| Take [2009] (39) | Ionizing irradiation | Radiation | Single dose | 8 Gy | 1 day | 40 mg/kg | IP |
| Zhang [2019] (40) | Chemical agent (Busulfan) | IP | Single dose | 30 mg/kg | 5 days | 100 mg/kg | IP |
| Abou-El-Naga [2021] (41) | Chemical agent (Busulfan) | IP | Single dose | 40 mg/kg | 30 days | 600 mg/kg | IP |
| Abd-El-Aziz [2012] (42) | Chemical agent (Busulfan) | IP | Single dose | 20 mg/kg | 5 days | 50 mg/kg | IP |
| Simsec [2008] (43) | Chemical agent (Cyclophosphamide) | Oral | Single dose | 100 mg/kg | 1 day | 20 mg/kg | IP |
| Supplementary table: study characteristics regarding the strategy of stress induction and melatonin therapy. IP, intraperitoneal; IV, intravenous; N/A, not available; Gy, Gray. | | | | | | | |

1. Wang Y, Zhao TT, Zhao HY, Wang H. Melatonin protects methotrexate-induced testicular injury in rats. Eur Rev Med Pharmacol Sci. 2018;22(21):7517-25.

2. Wang Z, Teng Z, Wang Z, Song Z, Zhu P, Li N, et al. Melatonin ameliorates paclitaxel-induced mice spermatogenesis and fertility defects. J Cell Mol Med. 2022;26(4):1219-28.

3. Yalçınkaya F, Gökçe A, Guven O, Davarcı M, Cikim G, Yekeler H, et al. N88 Protective effect of vitamine E and melatonin against radiation induced damage in testis of rat. European Urology Supplements - EUR UROL SUPPL. 2009;8:599-.

4. Zangoie R, Eshraghi H, Shirian S, Kadivar A, Nazari H, Aali E. Melatonin synergistically enhances protective effect of atorvastatin against busulfan-induced spermatogenesis injuries in a rat model. Comparative Clinical Pathology. 2020;29(1):161-6.

5. Zhang J, Fang Y, Tang D, Xu X, Zhu X, Wu S, et al. Activation of MT1/MT2 to Protect Testes and Leydig Cells against Cisplatin-Induced Oxidative Stress through the SIRT1/Nrf2 Signaling Pathway. Cells. 2022;11(10).

6. Zi T, Liu Y, Zhang Y, Wang Z, Wang Z, Zhan S, et al. Protective effect of melatonin on alleviating early oxidative stress induced by DOX in mice spermatogenesis and sperm quality maintaining. Reprod Biol Endocrinol. 2022;20(1):105.

7. Hussein MR, Abu-Dief EE, Abou El-Ghait AT, Adly MA, Abdelraheem MH. Melatonin and roentgen irradiation of the testis. Fertil Steril. 2006;86(3):750-2.

8. Khan S, Adhikari JS, Rizvi MA, Chaudhury NK. Radioprotective potential of melatonin against 60Co γ-ray-induced testicular injury in male C57BL/6 mice. Journal of Biomedical Science. 2015;22(1):61.

9. Kushwaha R, Nishad DK, Bhatnagar A, Khar RK. Melatonin-Caffeine Combination Modulates Gamma Radiation-induced Sperm Malformations in C57BL/6 Male Mice at Sublethal Dose of Gamma Radiation. J Pharm Bioallied Sci. 2021;13(2):268-75.

10. Lee K-M, Lee I-C, Kim S-H, Moon C, Park S-H, Shin D-H, et al. Melatonin attenuates doxorubicin-induced testicular toxicity in rats. Andrologia. 2012;44(s1):796-803.

11. Madhu P, Reddy KP, Reddy PS. Role of melatonin in mitigating chemotherapy-induced testicular dysfunction in Wistar rats. Drug Chem Toxicol. 2016;39(2):137-46.

12. Manda K, Bhatia AL. Prophylactic action of melatonin against cyclophosphamide-induced oxidative stress in mice. Cell Biol Toxicol. 2003;19(6):367-72.

13. Mirhoseini M, Saki G, Hemadi M, Khodadadi A, Mohammadi Asl J. Melatonin and testicular damage in busulfan treated mice. Iran Red Crescent Med J. 2014;16(2):e14463.

14. Moghadam MT, Dadfar R, Khorsandi L. The effects of ozone and melatonin on busulfan-induced testicular damage in mice. JBRA Assist Reprod. 2021;25(2):176-84.

15. Moradi M, Goodarzi N, Faramarzi A, Cheraghi H, Hashemian AH, Jalili C. Melatonin protects rats testes against bleomycin, etoposide, and cisplatin-induced toxicity via mitigating nitro-oxidative stress and apoptosis. Biomed Pharmacother. 2021;138:111481.

16. Cebi Sen C, Yumusak N, Atilgan HI, Sadic M, Koca G, Korkmaz M. The protective effect of melatonin on sperm quality in rat after radioiodine treatment. Andrologia. 2018;50(4):e12962.

17. Patil L, Balaraman R. Effect of Melatonin on Doxorubicin Induced Testicular Damage in Rats. International Journal of PharmTech Research CODEN( USA): IJPRIF ISSN. 2023;1:974-4304.

18. Aboelwafa HR, Ramadan RA, El-Kott AF, Abdelhamid FM. The protective effect of melatonin supplementation against taxol-induced testicular cytotoxicity in adult rats. Braz J Med Biol Res. 2022;55:e11614.

19. Alp BF, Kesik V, Malkoç E, Yiğit N, Saldır M, Babacan O, et al. The effect of melatonin on procarbazine induced testicular toxicity on rats. Syst Biol Reprod Med. 2014;60(6):323-8.

20. Ateşşahin A, Sahna E, Türk G, Ceribaşi AO, Yilmaz S, Yüce A, et al. Chemoprotective effect of melatonin against cisplatin-induced testicular toxicity in rats. J Pineal Res. 2006;41(1):21-7.

21. Baş E, Nazıroğlu M. Treatment with melatonin and selenium attenuates docetaxel-induced apoptosis and oxidative injury in kidney and testes of mice. Andrologia. 2019;51(8):e13320.

22. Chabra A, Shokrzadeh M, Naghshvar F, Salehi F, Ahmadi A. Melatonin ameliorates oxidative stress and reproductive toxicity induced by cyclophosphamide in male mice. Hum Exp Toxicol. 2014;33(2):185-95.

23. Cui Y, Ren L, Li B, Fang J, Zhai Y, He X, et al. Melatonin Relieves Busulfan-Induced Spermatogonial Stem Cell Apoptosis of Mouse Testis by Inhibiting Endoplasmic Reticulum Stress. Cell Physiol Biochem. 2017;44(6):2407-21.

24. Edrees Z, kader H, Embaby A, hameed E. The effect of melatonin on the testes of rats treated with cyclophosphamide: Histological and immunohistochemical study. The Egyptian Journal of Histology. 2012;35:822-32.

25. Kamal El-Dein EMKE-D, Anees LM. Ameliorative role of melatonin against cypermethrin or gamma irradiation induced testicular damage in male rats. International Journal of Radiation Research. 2020;18(4):765-76.

26. El-Shafaei A, Abdelmaksoud R, Elshorbagy A, Zahran N, Elabd R. Protective effect of melatonin versus montelukast in cisplatin-induced seminiferous tubule damage in rats. Andrologia. 2018;50(9):e13077.

27. Eren H, Mercantepe T, Tumkaya L, Mercantepe F, Dil E, Horsanali MO, et al. Evaluation of the protective effects of amifostine and melatonin against cisplatin induced testis injury via oxidative stress and apoptosis in rats. Experimental and Molecular Pathology. 2020;112:104324.

28. Filobbos S, Amin N, Yacoub M, Abd El_Hakim KR. Possible Protective Effect of Melatonin on Cisplatin-Induced Testicular Toxicity in Adult Albino Rats. A Histological and Immunohistochemical Study. Egyptian Journal of Histology. 2020;43(3):891-901.

29. Ghasemi FM, Faghani M, Khajehjahromi S, Bahadori M, Nasiri E E, Hemadi M. Effect of Melatonin on Proliferative Activity and Apoptosis in Spermatogenic Cells in Mouse under Chemotherapy. Journal of Reproduction and Contraception. 2010;21(2):79-94.

30. Ilbey YO, Ozbek E, Simsek A, Otunctemur A, Cekmen M, Somay A. Potential chemoprotective effect of melatonin in cyclophosphamide- and cisplatin-induced testicular damage in rats. Fertil Steril. 2009;92(3):1124-32.

31. Mohammad Ghasemi F, FaghaniLangroudi M, Falah Karkan M. The Protective Effect of Melatonin on Sperm Parameters, Epididymis and Seminal Vesicle Morphology in Adult Mouse Treated with Busulfan. Anatomical Sciences Journal. 2010;8(30):0-.

32. Ferdosi Khosroshahi A, Bakhtiari M, Soleimani Rad J, Koroji M, Roshangar L, Janzadeh A, et al. Study of the effect of exogenous melatonin on sperm fertility in busulfan induced oligospermic of pinealectomeized rat. Razi Journal of Medical Sciences. 2013;20(110):77-86.

33. فهیمه محمد ق, معصومه ف, سینا خواجه ج. اثر محافظتی ملاتونین بر تغییرات هیستولوژیک بیضه موش بالغ تحت درمان با بوسولفان. باروری و ناباروری. 1389;سال يازدهم(2):67-.

34. Olayaki LA, Adeyemi WJ, Adeyemi E, Osawaru O, Busura I, Jimoh S. Melatonin enhanced the restoration of biochemical profile in chlorambucil treated-rats: examination of after-withdrawal effects of the drug. Journal of African Association of Physiological Sciences. 2020;7(2):80-7.

35. Tawfik SS, Mansour HH, El-Shamy E, Sallam MH. Radioprotective Effect and Follow-up of Melatonin as Antifertility Drug in Male Adult Mice submitted to Whole-Body γ Irradiation. Egyptian Journal of Radiation Sciences and Applications. 2006;19(2):331-51.

36. Sukhorum W, Umka Welbat J, Krutsri S, Iamsaard Comma S. Protective effect of melatonin against methotrexate-induced testicular damage in the rat model: An experimental study. Int J Reprod Biomed. 2020;18(5):327-38.

37. Tajabadi E, Javadi A, Azar NA, Najafi M, Shirazi A, Shabeeb D, et al. Radioprotective effect of a combination of melatonin and metformin on mice spermatogenesis: A histological study. Int J Reprod Biomed. 2020;18(12):1073-80.

38. Torabi F, Malekzadeh Shafaroudi M, Rezaei N. Combined protective effect of zinc oxide nanoparticles and melatonin on cyclophosphamide-induced toxicity in testicular histology and sperm parameters in adult Wistar rats. Int J Reprod Biomed. 2017;15(7):403-12.

39. Take G, Erdogan D, Helvacioglu F, Göktas G, Ozbey G, Uluoglu C, et al. Effect of melatonin and time of administration on irradiation-induced damage to rat testes. Braz J Med Biol Res. 2009;42(7):621-8.

40. Zhang X, Xia Q, Wei R, Song H, Mi J, Lin Z, et al. Melatonin protects spermatogonia from the stress of chemotherapy and oxidation via eliminating reactive oxidative species. Free Radic Biol Med. 2019;137:74-86.

41. ABOU-EL-NAGA A-M, MOUSA S-A, ALTHOBAITI F, FAYAD E, FAHIM E-S. Ameliorative effects of melatonin and zinc oxide nanoparticles treatment against adverse effects of busulfan induced infertility in male albino mice. BIOCELL. 2022;46(2):535--45.

42. Abd El Aziz DH, Metwally HG. The effect of stem cell therapy versus melatonin on the changes induced by busulfan in the testes of adult rat: histological and immunohistochemical studies. Egyptian Journal of Histology. 2013;36(1):175-84.

43. Simsek A, Otunctemur A, Özcan L, Cilli M, Polat E, Somay A, et al. Preventive effects of melatonin in cisplatin and cyclophosphamide associated testes damage. European Urology Supplements - EUR UROL SUPPL. 2008;7:93-.
