# Supplementary material for: Protective effects of exogenous melatonin therapy against oxidative stress to male reproductive tissue caused by anti-cancer chemical and radiation therapy: a systematic review and meta-analysis of animal studies
Source: Front Endocrinol (Lausanne). 2023 Aug 28;14:1184745. doi: 10.3389/fendo.2023.1184745 (PMC10494246; doi:10.3389/fendo.2023.1184745)
Supplement: Supplementary file 1 [file DataSheet_1.zip › Supplementary Material/Supplementary Material 3.DOCX]

| 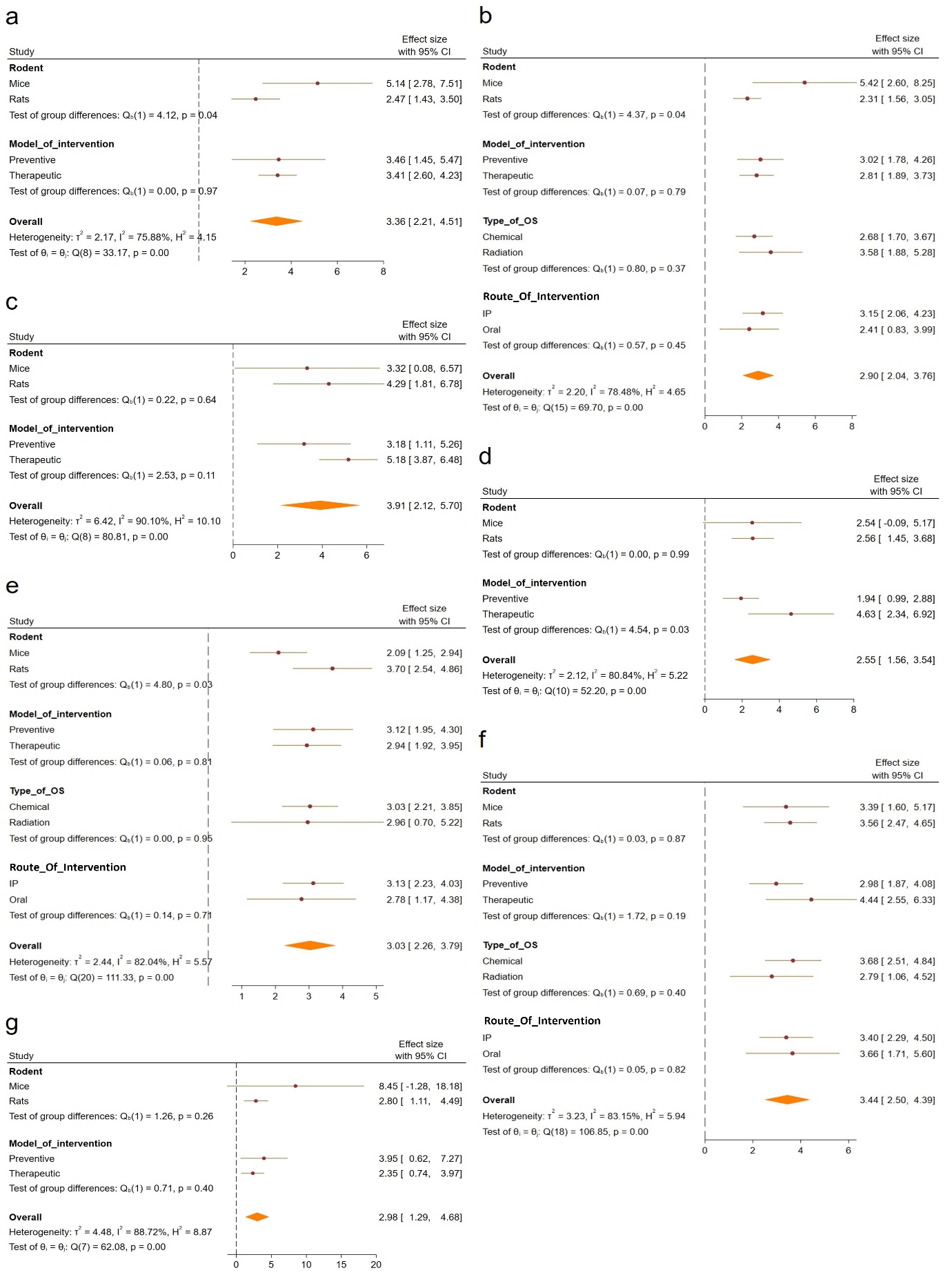 |
| --- |
| **Subgroup analyses: Sperm-related parameters including (a) JTBS, (b) normal sperm morphology, (c) number of spermatogonia, (d) seminiferous epithelial height, (e) seminiferous tubular diameter, (f) sperm count, and (g) sperm motility. JTBS: Johnsen's testicular biopsy score.** |
| 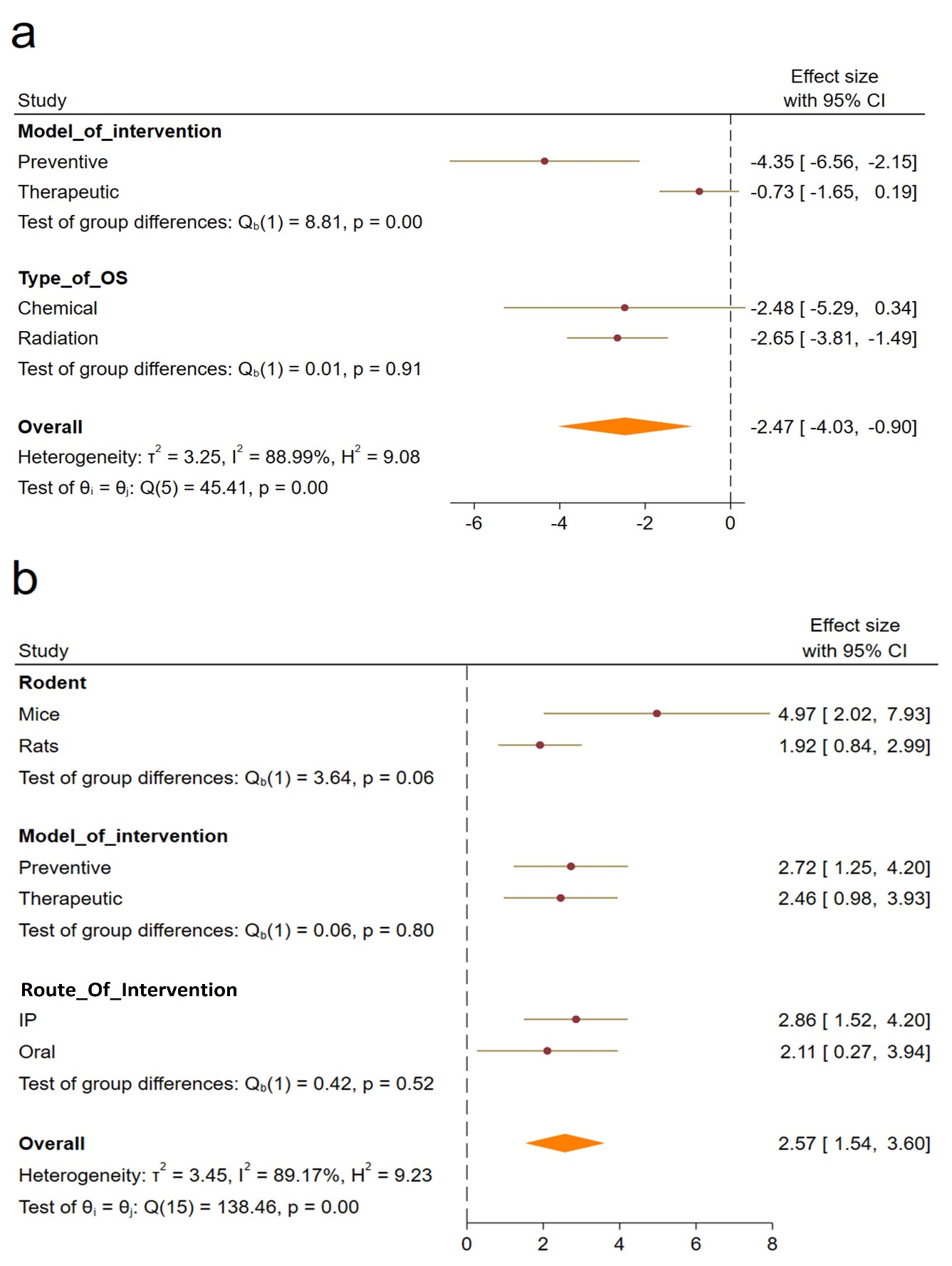 |
| **Subgroup analyses: Reproductive hormones including (a) serum FSH and (b) testosterone level. FSH: Follicle-Stimulating Hormone.** |

| 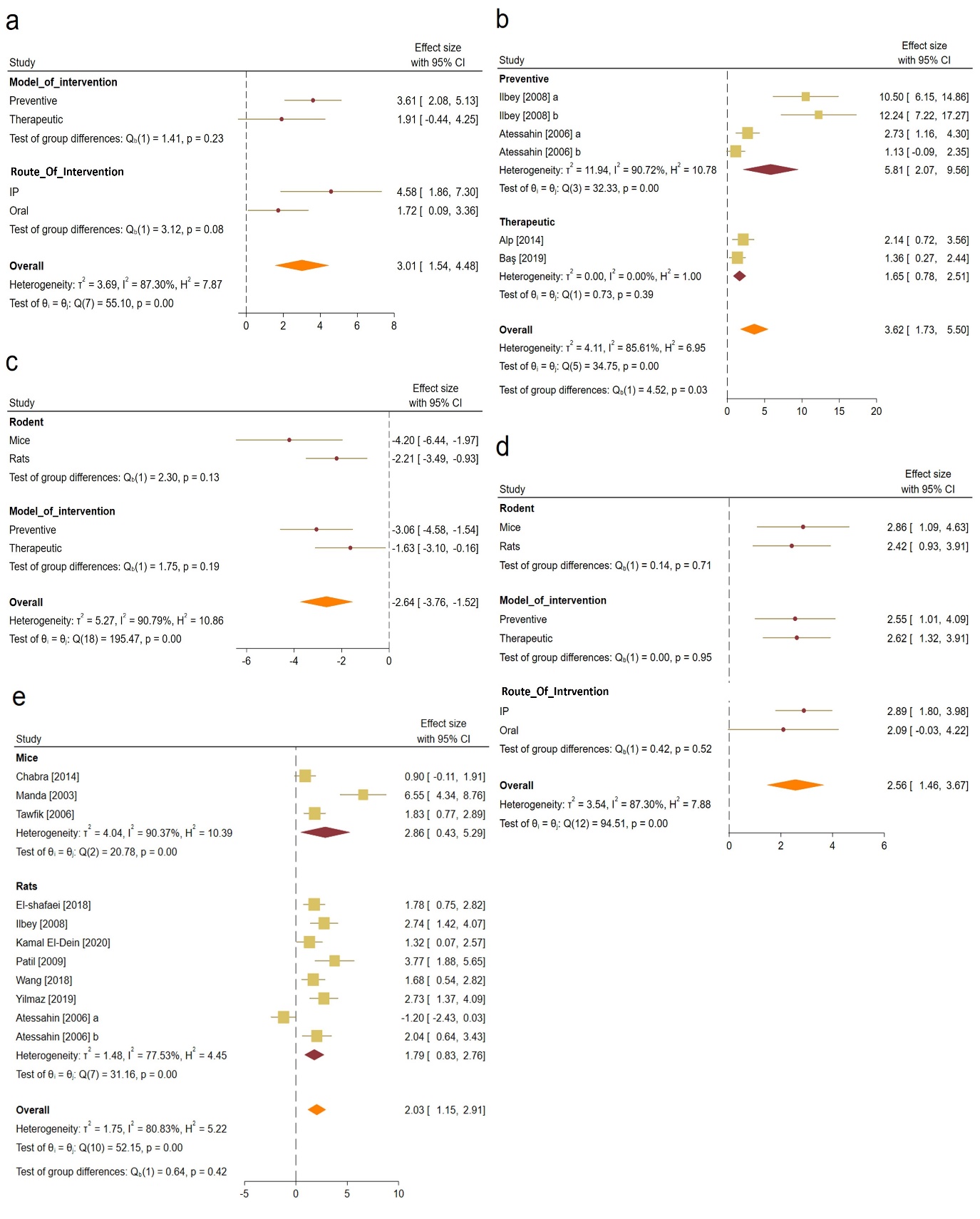 | |
| --- | --- |
| **Subgroup analyses: Testicular tissue’s oxidative markers including (a) caspase-3, (b) tissue CAT, (c) GPx, (d) MDA, (e) SOD activity, (f) TAC, and (g) GSH activity. CAT: catalase. GPx: glutathione peroxidase. MDA: malondialdehyde. SOD: superoxide dismutase, TAC: total antioxidant capacity, and GSH: glutathione.** | |
|  | |
| 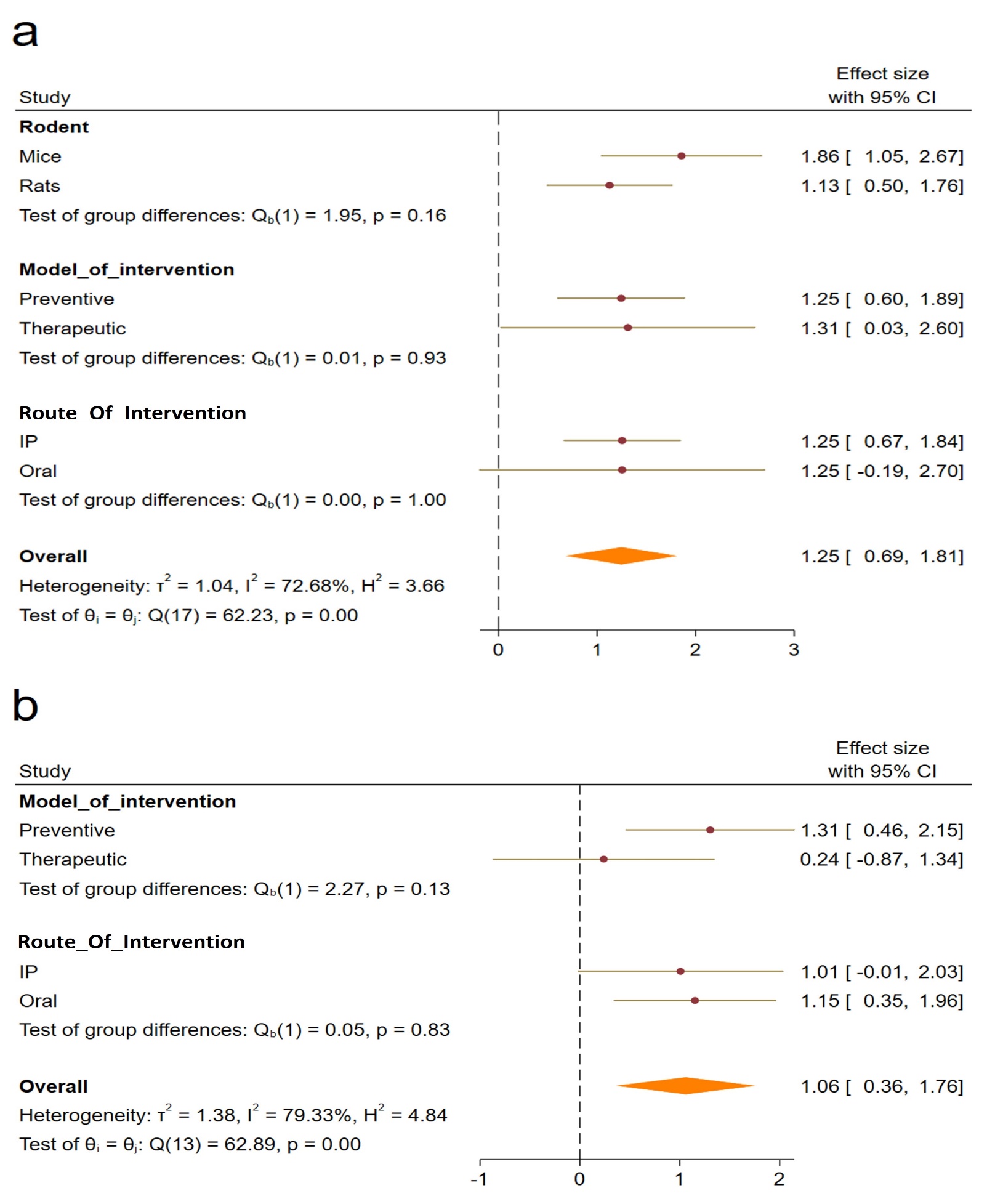 |  |
| **Subgroup analyses: Body and testicular weights including (a) absolute epididymis weight, (b) absolute testis weight, (c) body weight, and (d) testis to body relative weight** |  |
