# Supplementary material for: Protective effects of exogenous melatonin therapy against oxidative stress to male reproductive tissue caused by anti-cancer chemical and radiation therapy: a systematic review and meta-analysis of animal studies
Source: Front Endocrinol (Lausanne). 2023 Aug 28;14:1184745. doi: 10.3389/fendo.2023.1184745 (PMC10494246; doi:10.3389/fendo.2023.1184745)
Supplement: Supplementary file 1 [file DataSheet_1.zip › Supplementary Material/Supplementary Material 4.DOCX]

| 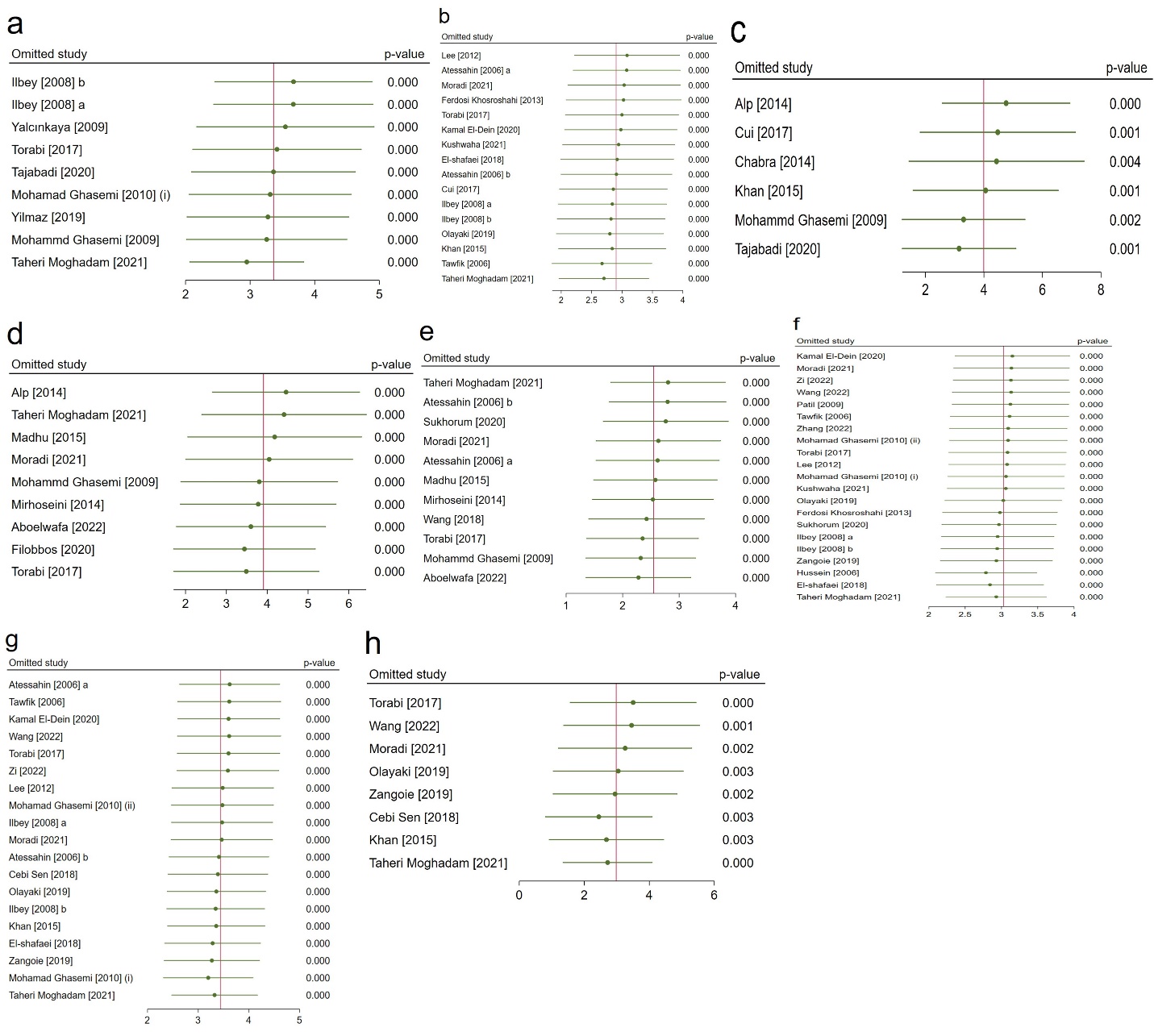 |
| --- |
| **Sensitivity analysis: Sperm-related parameters including (a) JTBS, (b) normal sperm morphology, (c) number of spermatogonia, (d) seminiferous epithelial height, (e) seminiferous tubular diameter, (f) sperm count, (g) sperm motility, and (h) sperm viability. JTBS: Johnsen's testicular biopsy score.** |

| 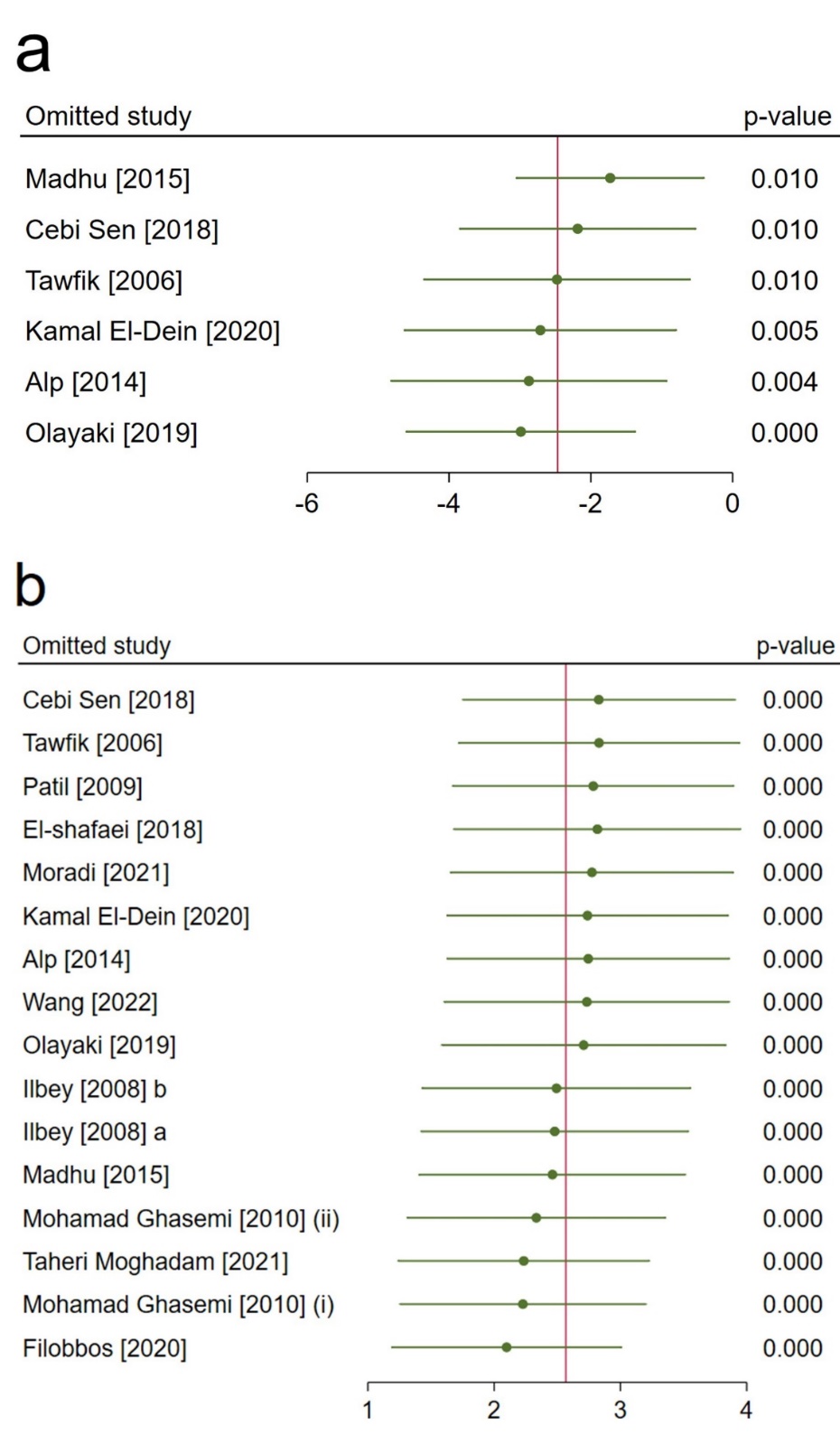 |
| --- |
| **Sensitivity analysis: Reproductive hormones including (a) serum FSH and (b) testosterone level. FSH: Follicle-Stimulating Hormone.** |
| 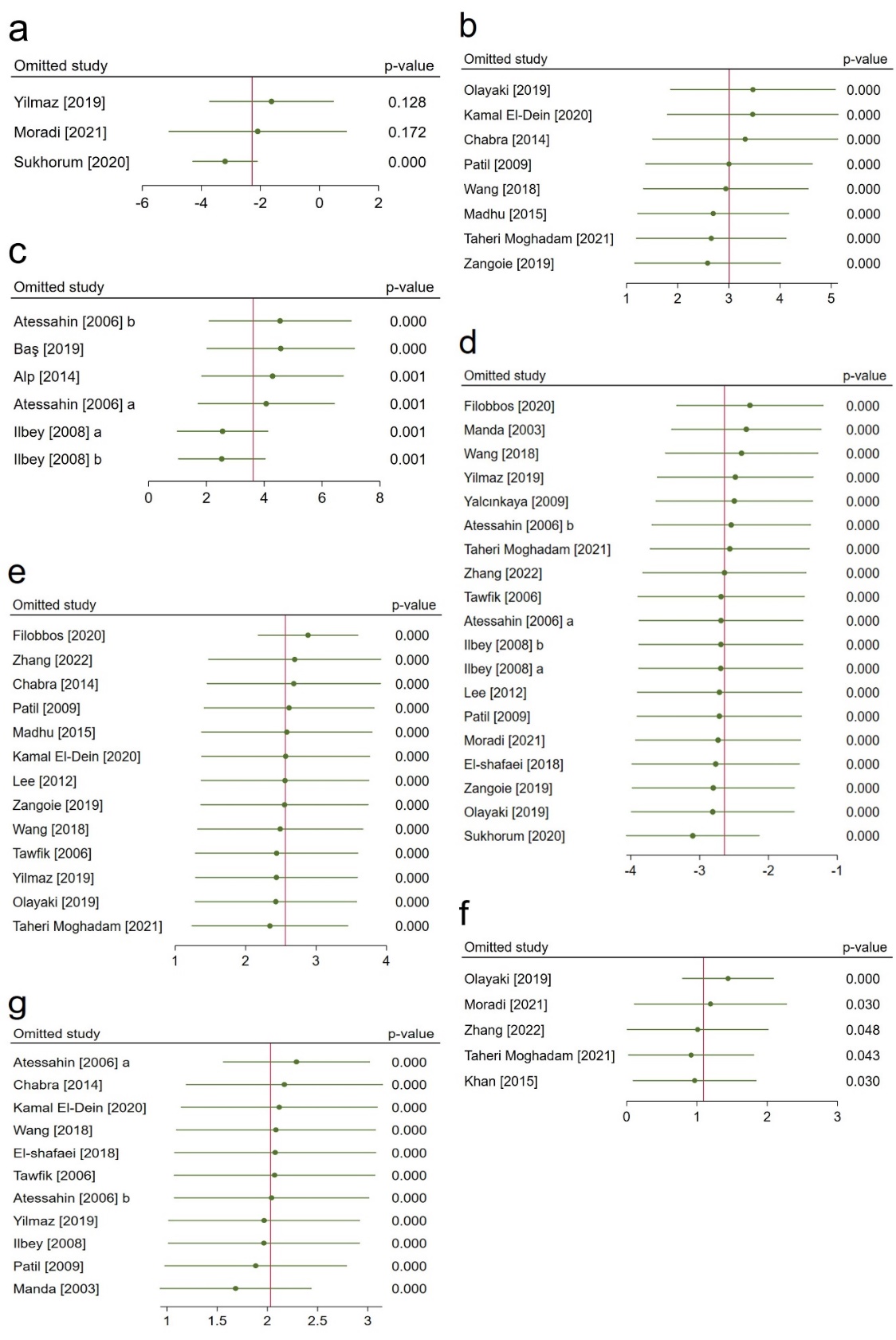 |
| **Sensitivity analysis: Testicular tissue’s oxidative markers including (a) caspase-3, (b) tissue CAT, (c) GPx, (d) MDA, (e) SOD activity, (f) TAC, and (g) GSH activity. CAT: catalase. GPx: glutathione peroxidase. MDA: malondialdehyde. SOD: superoxide dismutase, TAC: total antioxidant capacity, and GSH: glutathione.** |

| 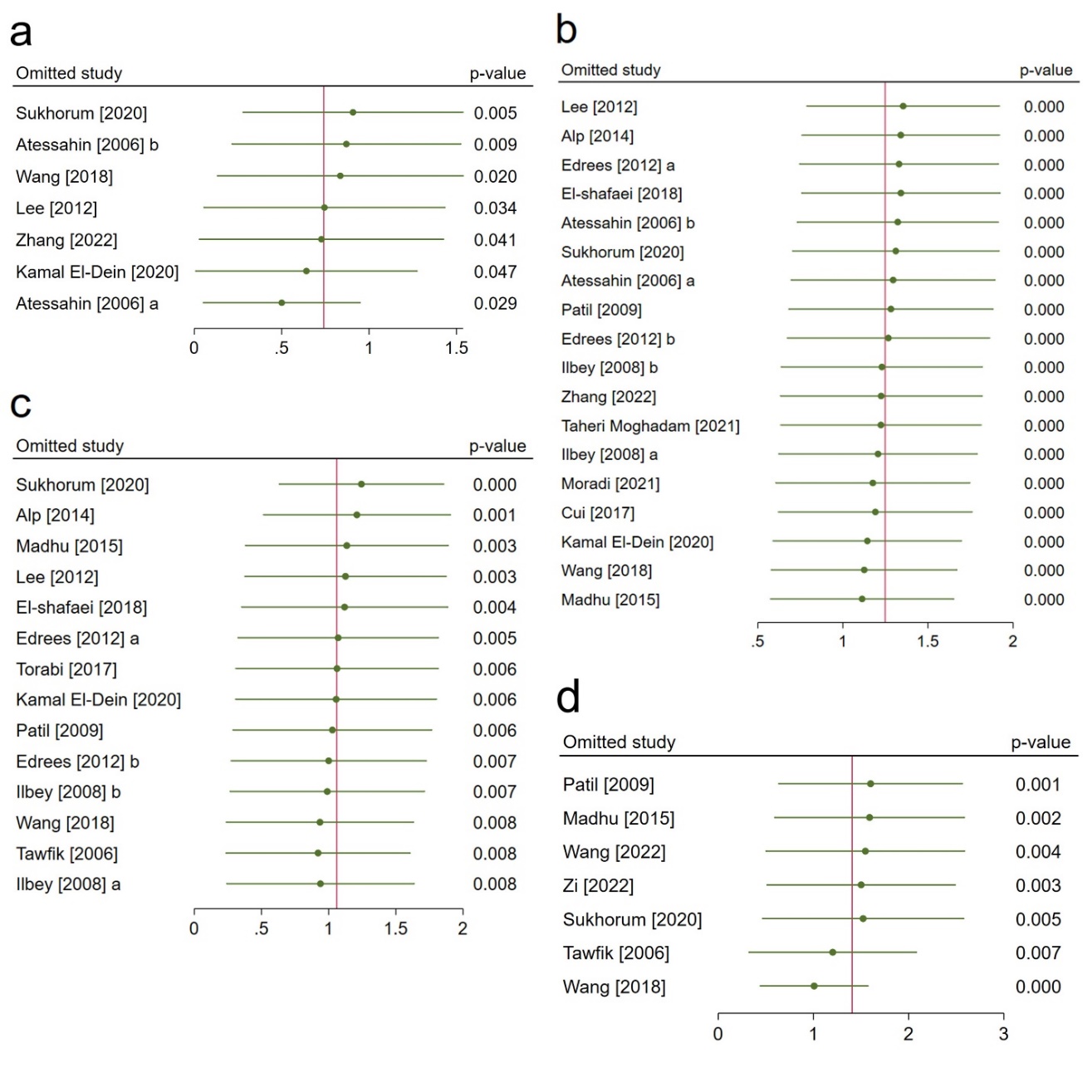 |
| --- |
| **Sensitivity analysis: Body and testicular weights including (a) absolute epididymis weight, (b) absolute testis weight, (c) body weight, and (d) testis to body relative weight.** |
