# Supplementary figures and images for: Protective effects of exogenous melatonin therapy against oxidative stress to male reproductive tissue caused by anti-cancer chemical and radiation therapy: a systematic review and meta-analysis of animal studies
Source: Front Endocrinol (Lausanne). 2023 Aug 28;14:1184745. doi: 10.3389/fendo.2023.1184745 (PMC10494246; doi:10.3389/fendo.2023.1184745)

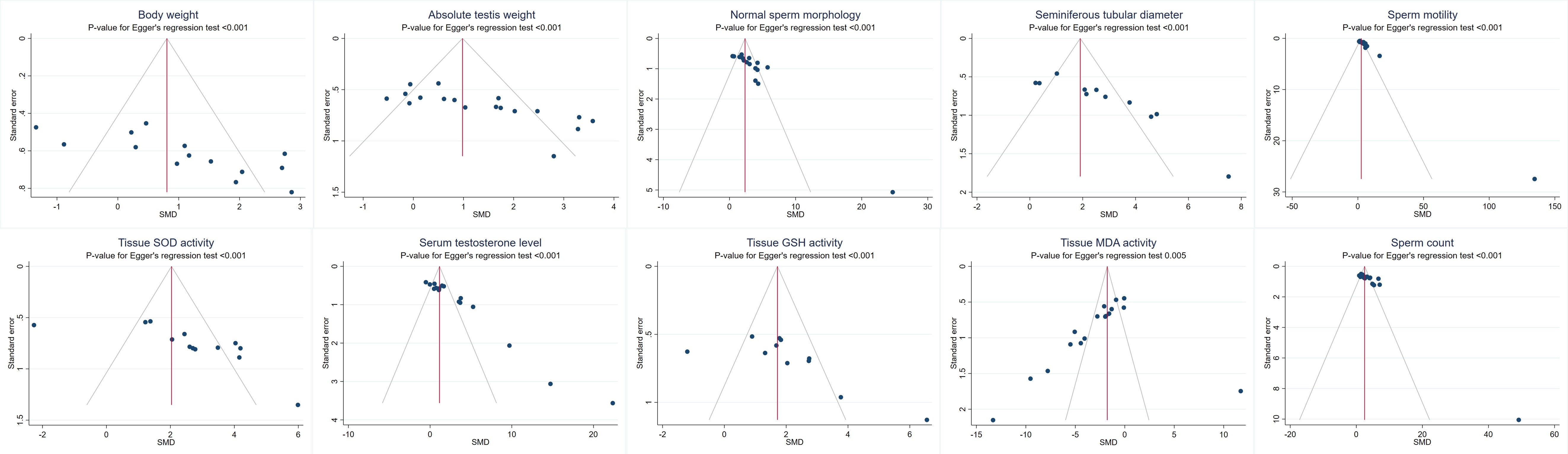

Supplement: Supplementary file 1 [file DataSheet_1.zip › Supplementary Material/Supplementary Material 6.JPEG]
